# Fast detection of differential chromatin domains with SCIDDO

**DOI:** 10.1093/bioinformatics/btaa960

**Published:** 2020-11-24

**Authors:** Peter Ebert, Marcel H Schulz

**Affiliations:** Institute for Medical Biometry and Bioinformatics, Heinrich Heine University, 40225 Düsseldorf, Germany; Max Planck Institute for Informatics, Saarland Informatics Campus, 66123 Saarbrücken, Germany; Max Planck Institute for Informatics, Saarland Informatics Campus, 66123 Saarbrücken, Germany; Cluster of Excellence on Multimodal Computing and Interaction, Saarland Informatics Campus, 66123 Saarbrücken, Germany; Institute for Cardiovascular Regeneration, Goethe University, 60590 Frankfurt am Main, Germany; German Center for Cardiovascular Research (DZHK), Partner site Rhein-Main, 60590 Frankfurt am Main, Germany

## Abstract

**Motivation:**

The generation of genome-wide maps of histone modifications using chromatin immunoprecipitation sequencing is a standard approach to dissect the complexity of the epigenome. Interpretation and differential analysis of histone datasets remains challenging due to regulatory meaningful co-occurrences of histone marks and their difference in genomic spread. To ease interpretation, chromatin state segmentation maps are a commonly employed abstraction combining individual histone marks. We developed the tool SCIDDO as a fast, flexible and statistically sound method for the differential analysis of chromatin state segmentation maps.

**Results:**

We demonstrate the utility of SCIDDO in a comparative analysis that identifies differential chromatin domains (DCD) in various regulatory contexts and with only moderate computational resources. We show that the identified DCDs correlate well with observed changes in gene expression and can recover a substantial number of differentially expressed genes (DEGs). We showcase SCIDDO’s ability to directly interrogate chromatin dynamics, such as enhancer switches in downstream analysis, which simplifies exploring specific questions about regulatory changes in chromatin. By comparing SCIDDO to competing methods, we provide evidence that SCIDDO’s performance in identifying DEGs via differential chromatin marking is more stable across a range of cell-type comparisons and parameter cut-offs.

**Availability and implementation:**

The SCIDDO source code is openly available under github.com/ptrebert/sciddo.

**Supplementary information:**

Supplementary data are available at *Bioinformatics* online.

## 1 Introduction

Large epigenome mapping consortia, such as DEEP (www.deutsches-epigenom-programm.de), BLUEPRINT (Adams *et al.*, 2012) or ENCODE (The ENCODE Project Consortium *et al.*, 2012) produce an ever-increasing amount of reference epigenomes for a multitude of different cell types. With the ultimate goal of compiling a publicly available catalog of 1000 reference epigenomes released under the IHEC umbrella (http://ihec-epigenomes.org), the computational interpretation of large amounts of epigenome data presents a formidable challenge for bioinformatics. However, the cell-type specific and dynamic nature of the epigenome adds substantial complexity to the problem of characterizing cellular similarities and differences on the epigenetic level. Moreover, limited resources commonly force scientists to investigate only a small number of biological replicates per condition of interest. Despite all these challenges, the discoveries in the field of epigenomics have greatly enhanced our understanding of transcriptional regulation, cellular identity and disease development (Bernstein *et al.*, 2007; Hemberger *et al.*, 2009; Jones and Baylin, 2007; Ladewig *et al.*, 2013; Lowdon *et al.*, 2016).

An important component of the epigenetic landscape is post-translational modifications of histone proteins, briefly referred to as histone marks. The interpretation of histone mark data is particularly intricate as the interplay between different histone marks results in a combinatoric complexity that is largely absent for other epigenetic modifications, such as DNA methylation. For example, bivalent chromatin domains that mark developmental genes in embryonic stem cells represent a biologically meaningful co-occurrence of several different histone marks (Bernstein *et al.*, 2006). The realization that histone mark combinations can be interpreted as local activity states of the genome, so-called chromatin states, led to the widespread use of probabilistic graphical models to discover these ‘hidden states’ (Ernst *et al.*, 2011; Ernst and Kellis, 2012; Hoffman *et al.*, 2012; Mammana and Chung, 2015; Song and Chen, 2015). Popular tools, such as ChromHMM (Ernst and Kellis, 2012) or EpiCSeg (Mammana and Chung, 2015), have tremendously simplified the analysis of histone data as they summarize the combined effect of histone mark co-occurrences in a manageable number of discrete chromatin states. After functional characterization, the discovered chromatin states are commonly augmented with textual labels to ease interpretation, e.g. labeling regions as active or poised promoters, or distinguishing between weak and strong transcriptional activity. However, in our experience, the generated chromatin state maps are often manually inspected in only a limited number of loci, or simply serve as additional genomic annotation data. Given that chromatin state maps provide a neat abstraction of the various histone mark combinations, it stands to reason that a more comprehensive view on them may offer valuable guidance in exploratory studies.

Given the large variety of tools for differential histone mark analysis, it is not surprising that different design choices may limit a tools’ applicability in certain use cases. For example, PePr (Zhang *et al.*, 2014) can scan for differential histone marking in replicated experiments and in an unbiased, i.e. genome-wide manner, but has no inherent support for histone mark combinations. Other tools, e.g. dPCA (Ji *et al.*, 2013), can analyze several histone marks in combination, but do so only in predefined regions, such as promoters, which biases the analysis to the local genomic context.

When focusing on the differential analysis of chromatin state maps, we found that there are only few tools available, and some exhibit similar limitations as mentioned above. ChromDet (Carrillo-de Santa-Pau *et al.*, 2017) can be applied in a genome-wide manner and uses Multiple Correspondence Analysis (an analog to Principal Component Analysis for categorical data) followed by an iterative clustering approach to identify regions that perfectly partition the samples into cell-type or lineage-specific groups (so-called chromatin determinant regions). The computational burden of a ChromDet analysis is lowered by various filtering steps to remove uninformative or outlier regions, which renders ChromDet analyses prohibitive for small sample numbers where limited inter-group variance leads to a substantial amount of dropped regions. This preprocessing also requires enough insight into the nature of the samples at hand to manually set appropriate filtering thresholds.

Other available tools for the differential analysis of chromatin state maps are limited to the analysis of a predefined set of genomic regions. The ChromDiff (Yen and Kellis, 2015) tool represents chromatin states in user-specified regions of interest, e.g. the bodies of all coding genes, as normalized coverage vectors. ChromDiff uses the Mann–Whitney–Wilcoxon test to identify differential chromatin states between sample groups, e.g. contrasting all male and female samples. Because ChromDiff relies on standard statistical tests, sufficient statistical power in terms of number of available samples is mandatory to find any significant differences between the groups. The recently published Chromswitch package (Jessa and Kleinman, 2018) similarly identifies differential chromatin states only in preselected regions of interest. Chromswitch can only analyze a single chromatin state at a time and uses a binary ‘presence/absence’ encoding to construct feature vectors that are subsequently clustered. The cluster assignments resulting from the hierarchical clustering are then scored by their agreement with the known biological labels of the samples and manual thresholding on these scores is required to select the final set of chromatin state switches.

A common denominator of all existing methods is that they consider chromatin state similarity as a binary variable, i.e. any chromatin state is, to exactly the same extent, (dis-) similar to any other chromatin state. We argue that this is an oversimplification and, as we will show below, much is to be gained when measuring chromatin state similarity using a quantitative scale.

In summary, current methods are limited to region-based analysis, focus on individual chromatin states, require a comparatively large number of biological replicates for their statistical analysis, and use a rather basic representation of chromatin state similarity, which hinders general applicability of existing methods.

We devised a new method for the score-based identification of differential chromatin domains (SCIDDO) with the goal of providing a generally applicable tool for the fast identification of differential chromatin marking. One of SCIDDO’s main features is its capability to identify potentially large and heterogeneous regions of differential chromatin marking, which we refer to as differential chromatin domains (DCDs). The statistical evaluation of the identified domains relies on well-established theory borrowed from score-based biological sequence analysis. The borrowed theory enables an interpretable presentation of SCIDDO’s results and facilitates downstream analysis. In the following, we present results from analyzing four replicated sample groups, highlighting the useful biological interpretability of the identified DCDs.

## 2 Materials and methods

### 2.1 Experimental data overview

We used chromatin immunoprecipitation sequencing (ChIP-seq) (H3K4me1, H3K4me3, H3K27ac, H3K27me3, H3K36me3 and H3K9me3) and RNA-seq data of the following replicated DEEP samples for our analysis: HepG2 (HG 1 and 2), hepatocytes (He 2 and 3), Mo 1, 3 and 5 (Wallner *et al.*, 2016) and macrophages [Ma 3 and 5 (Wallner *et al.*, 2016)]. Please refer to the Supplementary Methods for complete information on the experimental data used in this study.

### 2.2 Data preprocessing

All histone, gene expression and genomic annotation data were preprocessed following standard procedures, e.g. as recommended in the help pages of the respective software tool. Detailed descriptions of the preprocessing steps including parameter settings are given in the Supplementary Methods.

### 2.3 Statistical background for SCIDDO

The theory behind the statistical evaluation available in SCIDDO has been developed in the context of biological sequence analysis, e.g. to identify runs of hydrophobic amino acids in protein sequences (Karlin and Altschul, 1990; Karlin *et al.*, 1990). Since the theory was left unaltered, we give only a compact overview to introduce the necessary concepts and nomenclature. The chromatin state maps of each sample in the SCIDDO dataset can be represented as a sequence $X=\{x_{1}\ldots x_{p}\ldots x_{n}\}$. Here, the *x_p_* are assumed to be i.i.d. random variables over an alphabet *A* and *n* is the length of the sequence. In our case, |A|=18 representing the 18 different chromatin states of the ChromHMM Hidden Markov Model (CMM18; see Supplementary Methods). Each pair of states $\left(a^{i},a^{j}\right)$ is assigned a score *s^ij^*, where $s^{ij}<0$ indicates state similarity (uninteresting regions) and $s^{ij}>0$ indicates state dissimilarity (interesting regions; see below for derivation of the *s^ij^*). We omit the superscript *ij* in the following to improve readability. When comparing two chromatin state maps *X*, *Y*, each state pairing (*x_p_*, *y_p_*) is assigned the respective score *s* as defined above. This results in a sequence of scores $S=\{s_{1}\ldots s_{n}\}$ that is scanned for subsegments of highest cumulative score. This approach is called local score computation and can be done efficiently with a linear time algorithm (Ruzzo and Tompa, 1999). The set of all maximal scoring disjoint segments returned by this algorithm represents the set of candidate regions for the respective chromatin state map comparison. The (unnormalized) raw score *R* of a candidate region is defined as the sum over all scores in the candidate region $R=\sum_{k\leq p\leq l}s_{p}$, where *k* and *l* indicate the position of the leftmost and of the rightmost genomic bin included in the candidate region. These cumulative scores have to be normalized to account for the fact that higher scores have a higher chance of occurring with increasing sequence length. This normalization step requires the estimation of two statistical parameters *λ* and *K* [for detailed derivation of these parameters, see Karlin *et al.* (1990)]. Since both *λ* and *K* lack a biologically meaningful interpretation, they can be simply thought of as scaling factors for the scoring system and the search space. For this parameter estimation, SCIDDO relies on the routines implemented in BLAST v2.7.1 (Altschul *et al.*, 1990). Additionally, four assumptions are needed for the theory to be applicable, which then enables modeling the limiting behavior of the score distribution as Gumbel-type extreme value distribution [see Karlin *et al.* (1990)]:

1. The sequences are infinitely long
2. The *x_p_* are i.i.d. random variables
3. A positive score must be possible
4. The expected score is negative.

Assumptions 1 and 2 of course do not apply to any biological sequence, but are needed for reasons of mathematical tractability (Karlin *et al.*, 1990). Assumptions 3 and 4 are tested by SCIDDO before starting the actual analysis, safeguarding against errors in the statistical evaluation. Under these conditions, the Expect value (*E*) for a DCD with raw score *R* is then calculated as

(1) $$E=K\cdot L\cdot e^{-\lambda R},$$

where the factor *L* is the length of the chromosomal sequence adapted for replicate-variation. Since SCIDDO has been designed to compare (small) groups of replicates against each other, we adapted the calculation of the total length of the sequence. Intuitively, adding more and more biological replicates to a group of samples does not linearly increase the amount of information contained in the respective group. At some point, all biologically meaningful variation has been sampled and, ignoring technical artifacts and stochastic effects, no new chromatin states should be observed at any position of the genome. Based on this consideration, for each additional replicate in a group of samples, SCIDDO only adds those positions to the total sequence length that show a new chromatin state compared to all other biological replicates already in the group. A complete description of this computation is given as pseudocode in the Supplementary Methods (Algorithm 1).

We note that, it seems difficult to test our above claim directly since, to the best of our knowledge, there is no high-quality ChIP-seq dataset publicly available that consists of a large number (say, 10 or more) of biological replicates for all six histone marks. However, given that the cell-type specificity of chromatin marking is largely uncontested, it seems compelling that the amount of natural variation at the chromatin level has to be limited to ensure phenotype stability.

### 2.4 Derivation of pairwise chromatin state similarity scores

The theoretical considerations presented in the previous section do not enforce the use of complicated scoring systems that are well-grounded in theory, e.g. rather simple ‘match/mismatch’ or empirically derived scoring systems can be used if considered appropriate (Karlin and Altschul, 1990). We thus decided to use the emission probability vectors of the 18 chromatin states (= the hidden states of the ChromHMM Hidden Markov Model) to compute pairwise similarity scores. The state emissions $E_{i}=\left(e_{i}^{h}\ldots e_{i}^{h}\right)$ for state *a_i_* represent a probability distribution over the observed outputs, i.e. over the observed six histone modifications *h*. This motivated using the symmetric Jensen–Shannon divergence (JSD) (Lin, 1991) to compute chromatin state similarities

(2) $$\mathrm{JSD}(E_{i},E_{j})=2\cdot H\left(\frac{E_{i}+E_{j}}{2}\right)-H\left(E_{i}\right)-H\left(E_{j}\right),$$

where *H* is the Shannon entropy

(3) $$H(E_{i})=-\sum_{h=1}^{6}e_{i}^{h}\cdot \;\text{log}(e_{i}^{h}).$$

Since the JSD has a lower bound of 0, the pairwise similarities for each state were shifted by subtracting the mean JSD. This resulted in negative scores for similar states (JSD near zero) and positive scores for dissimilar states. Scores are commonly represented by integer values, which we realized by multiplying the real-valued scores by a factor of 10 and rounding them to integers afterwards. As mentioned above, SCIDDO checks the adherence to Assumptions 3 and 4 for any custom scoring scheme, such as our JSD-derived one to ensure applicability of the Karlin–Altschul statistics (Karlin and Altschul, 1990; Karlin *et al.*, 1990) before starting a differential analysis.

A peculiarity of chromatin state maps is the so-called background state (state 18 labeled as ‘quiescent’ in the CMM18 model). This state represents the lack of any detectable signal in the input data. As it is *a priori* impossible to identify the true source for this lack of a signal, i.e. it could be a technical artifact or biologically meaningful, the background state needs to be handled with special care in the interpretation of chromatin state maps. We decided to implement a cautious strategy and replaced all pairwise state similarities involving the background state with the minimal score generated with our JSD-based approach. In other words, the background state is similar, i.e. not differential relative to all other chromatin states. We opted for this strategy to avoid finding DCDs that are dominated by the background state and could thus be challenging to interpret.

### 2.5 Availability of raw data and code

Access to the raw DEEP sequencing data can be requested under www.ebi.ac.uk/ega/dacs/EGAC00001000179.

Pipeline code to reproduce all results and figures of this study is available under doi.org/10.17617/1.6K. The Python source code of the SCIDDO tool is openly available under github.com/ptrebert/sciddo.

## 3 Results

### 3.1 Score-based identification of differential chromatin domains

**Fig. 1.**
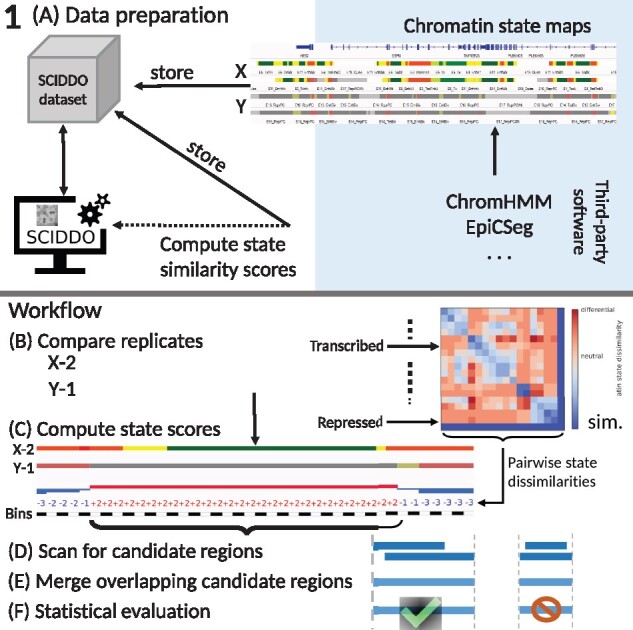
Overview of SCIDDO’s workflow to identify DCDs: (**A**) Data preparation: chromatin state maps can be generated using common tools (blue shaded area). The chromatin state maps for all replicates of sample groups X and Y are stored together with the chromatin state emission probabilities in a SCIDDO dataset to ensure later reproducibility of the analysis. The state emission probabilities are used to compute chromatin state similarity scores. (**B**)–(**F**) Workflow: the differential analysis starts by comparing all replicate pairs in the dataset, here exemplified as X-2 versus Y-1 (B). All observed chromatin state pairs are scored with their respective dissimilarity score (C). The resulting score sequences are scanned for high-scoring candidate regions (D). Overlapping candidate regions of all replicate pairs are then merged (E) and filtered after statistical evaluation to generate the final set of DCDs (F). diff., high scores indicate differential chromatin states; sim., low scores indicate similar chromatin states

The differential analysis with SCIDDO consists of two major parts, data preparation and the actual analysis run (see Fig. 1 for an overview). In the data preparation step (Fig. 1A), SCIDDO creates a single coherent dataset storing all data and metadata relevant for the analysis to ensure later reproducibility of the results. During data preparation, the state emission probabilities of the chromatin state segmentation model are used to compute pairwise chromatin state dissimilarities (see Section 2). SCIDDO then performs the differential analysis as follows: for each comparison contrasting sample group X versus group Y, SCIDDO compares individual replicates against each other, say, X-2 versus Y-1 (Fig. 1B). Each observed chromatin state pair in the two chromatin state maps is assigned a score that quantifies the dissimilarity of the two states: positive scores indicate state dissimilarity, and negative scores indicate state similarity (Fig. 1C; see Section 2). Candidate regions for differential chromatin marking are identified on this level of replicate comparisons by searching for chromosomal segments that show a high cumulative score, which indicates a strong dissimilarity on the chromatin state level; hence, we refer to this value as the differential chromatin score (DCS) of the segment (Fig. 1C and D). Extracting segments based on (locally) maximal DCSs implies also a maximization of the segment length, and no minimum or maximum length has to be specified. To proceed from candidate regions identified in individual replicate comparisons (e.g. X-2 versus Y-1) to candidate regions that are representative of all samples X versus Y, overlapping candidate regions are merged by averaging their DCSs and taking the union of their genomic coverages (Fig. 1E). Finally, the segment DCSs are turned into an Expect (*E*) value, which allows to filter the resulting candidate regions for their statistical significance (Fig. 1F). The *E*-value (see Section 2) has the interpretation of indicating how many candidate regions with at least a similarly high DCS could arise simply due to chance when comparing random sequences of the same length. In other words, filtering the candidate regions for a default *E*-value of <1 to call DCDs restricts the results to those regions where the chromatin states are so different between the samples that one would not expect to find such a difference simply due to chance. To simplify visualizations, we report *E*-values after a negative log10 transform in the remainder of this study. The aforementioned threshold of one is thus transformed to zero and larger *E*-values indicate higher statistical stringency.

To demonstrate the usefulness of SCIDDO in a differential analysis setting, we compiled a medium-sized dataset of high-quality DEEP samples that includes both distantly as well as more closely related cell types (see Section 2). Specifically, there is only a single step of cellular differentiation separating monocytes (Mo 1, 3 and 5) from macrophages (Ma 3 and 5). The liver cell line HepG2 (HG 1 and 2) is commonly used as an *in vitro* model in liver-related studies, but its state as an immortalized cell line distinguishes it from the primary hepatocytes (He 2 and 3) in our dataset. Hence, the dataset we compiled enabled us to evaluate SCIDDO’s performance at various degrees of ‘cellular relatedness’.

We used SCIDDO to perform a differential analysis for all six possible pairings of sample groups in our dataset: (i) HepG2 [HG] versus hepatocytes [He]; (ii) HepG2 versus monocytes [Mo]; (iii) HepG2 versus macrophages [Ma]; (iv) hepatocytes versus monocytes; (v) hepatocytes versus macrophages and (vi) monocytes versus macrophages.

The entire analysis including SCIDDO’s internal data preparation (Fig. 1A) completed within minutes on a moderately powerful compute server (Supplementary Table S4). Additionally, we confirmed that our data follow the theoretical assumptions and that the estimated statistical parameters are within reasonable boundaries (see Section 2 and Supplementary Result S2.1). Due to the robust identification of DCDs between individual replicates (Supplementary Result S2.2), and the consistent patterns of chromatin state switches in DCDs (Supplementary Result S2.3), the results presented in the following take a biological perspective relative to the four cell types in our dataset, and we usually omit considerations involving only the replicates of one cell type.

### 3.2 DCDs occur in various regulatory contexts

**Fig. 2.**
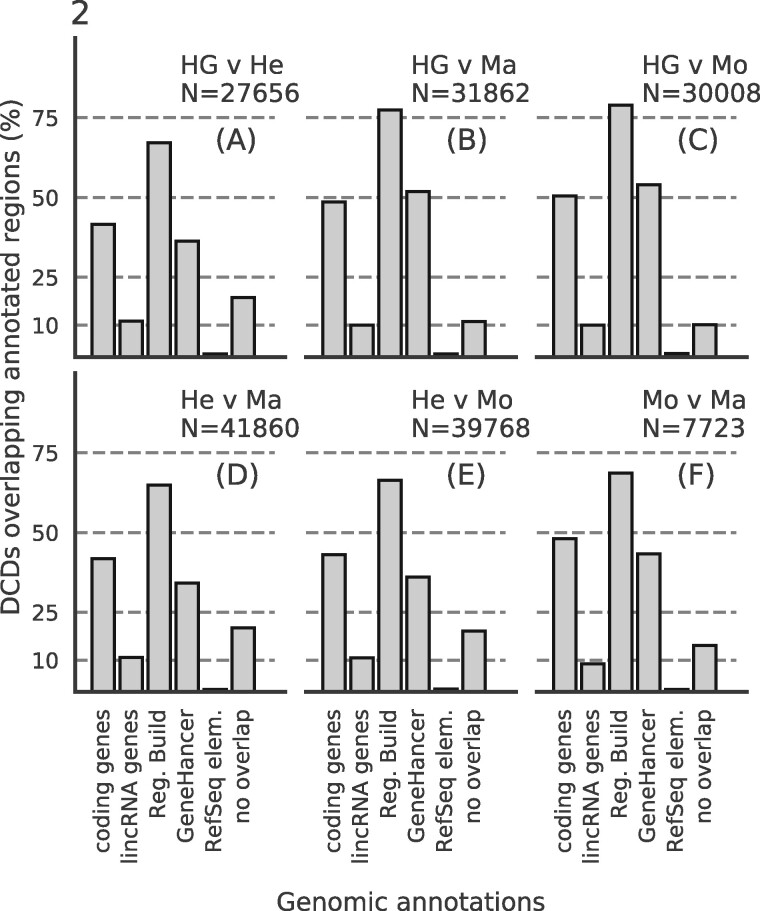
DCDs overlap with annotated regulatory regions: bar heights indicate percentage of identified DCDs that overlap with different genomic annotations for all six sample group comparisons: (**A**) HepG2 [HG] versus hepatocytes [He]; (**B**) HepG2 versus macrophages [Ma]; (**C**) HepG2 versus monocytes [Mo]; (**D**) hepatocytes versus macrophages; (**E**) hepatocytes versus monocytes; (**F**) monocytes versus macrophages. *N*, total number of identified domains; coding genes, Gencode v21 protein-coding genes; lincRNA genes, Gencode v21 lincRNA genes; Reg. Build, Ensembl Regulatory Build v78; GeneHancer: GeneHancer annotated enhancers limited to Gencode v21 gene set; Refseq elem., RefSeq functional elements

Since it is well established that histone marks occur in various regulatory contexts, e.g. ranging from promoters and enhancers to gene bodies, it stands to reason that *bona fide* DCDs should predominantly occur in similar regulatory contexts. To test this hypothesis, we intersected the DCDs identified by SCIDDO with various annotation datasets and observed that around 80–90% of all DCDs overlap with at least one type of genomic annotation (Fig. 2). Since there is no theory that would enable us to formulate an *a priori* expectation about the extent to which differences on the chromatin level should occur between any two cell types, we cannot assess the plausibility of the absolute numbers of identified domains. Nevertheless, it can be observed that the lowest number of domains is detected in the comparison of monocytes to macrophages (Fig. 2F), i.e. when comparing the two most closely related cell types in our dataset. For all other comparisons, the number of identified chromatin domains is ∼4- to more than 5-fold higher, but yet shows a similar tendency of a smaller number of identified chromatin domains for more closely related cell types.

We also examined if there was a notable difference in the magnitude of the *E*-value for DCDs overlapping regulatory regions compared to DCDs without such an overlap. We found that to be the case (Supplementary Result S2.4), which suggests that selecting DCDs for more in-depth analysis based on the magnitude of the *E*-value could be a viable strategy.

### 3.3 Formation of DCDs affects gene expression

The results presented in the previous section indicate that DCDs largely overlap with a variety of regulatory regions, and thus it seems plausible that the formation of a DCD should have functional consequences, e.g. by modulating gene expression levels. Apart from basic considerations about the magnitude of the observed *E*-values, we also hypothesized that DCDs covering larger parts of the gene body could indicate stronger changes in gene expression. To give a canonical example, a gene that is entirely repressed by means of polycomb-mediated silencing should be enriched for the histone mark H3K27me3, and this marking should be replaced by H3K36me3 as soon as the gene is activated and actively transcribed (Barski *et al.*, 2007). On the other hand, if the gene expression is modulated, e.g. by changing transcription factor binding in enhancer regions, the effect on the chromatin marking in the gene body could arguably be less pronounced. To investigate this hypothesis, we stratified all genes by the fraction of their gene body length being covered by a DCD (no overlap in gene body or enhancers, less or more than 50% gene body overlap). Next, we computed gene expression fold changes using DESeq2 (Love *et al.*, 2014) (see Supplementary Methods) for the six sample group comparisons and visualized the fold change for all genes in the three DCD overlap groups as a cumulative distribution (Supplementary Figs S7–S9). The curves indicate that genes covered by more than 50% of their body length with a DCD indeed exhibit stronger changes in their expression level (orange lines). A similar effect, albeit weaker, can be observed for genes having <50% of their body or their promoter covered by a DCD (blue lines). In many cases, the difference in fold change relative to the group of genes that does not overlap a DCD is significant. Additionally, we applied the same method to test if the number of gene-associated enhancers that overlap a DCD had a similar bearing on gene expression (Supplementary Figs S7–S9, middle and right panels). This enhancer-centric view shows a stable pattern across most sample comparisons indicating that stronger changes in gene expression occur if more gene-associated enhancers overlap a DCD. This observation is particularly intriguing when restricting the view on intergenic enhancers, where, as opposed to intragenic enhancers, there is lower chance of a coincidental overlap with a DCD. In general, a small but noticeable difference compared to the no DCD overlap group (gray dashed line) can be expected as soon as 2–3 enhancers show a DCD (magenta curve).

### 3.4 SCIDDO detects chromatin changes in differentially expressed genes

SCIDDO does not impose any restrictions on the regions of interest that can be interrogated in a differential analysis. Since there is no general model of chromatin variation that would enable us to assess the plausibility of the identified DCDs irrespective of their genomic context, we decided to focus on a small-scale case study that is arguably of broad biological interest.

**Fig. 3.**
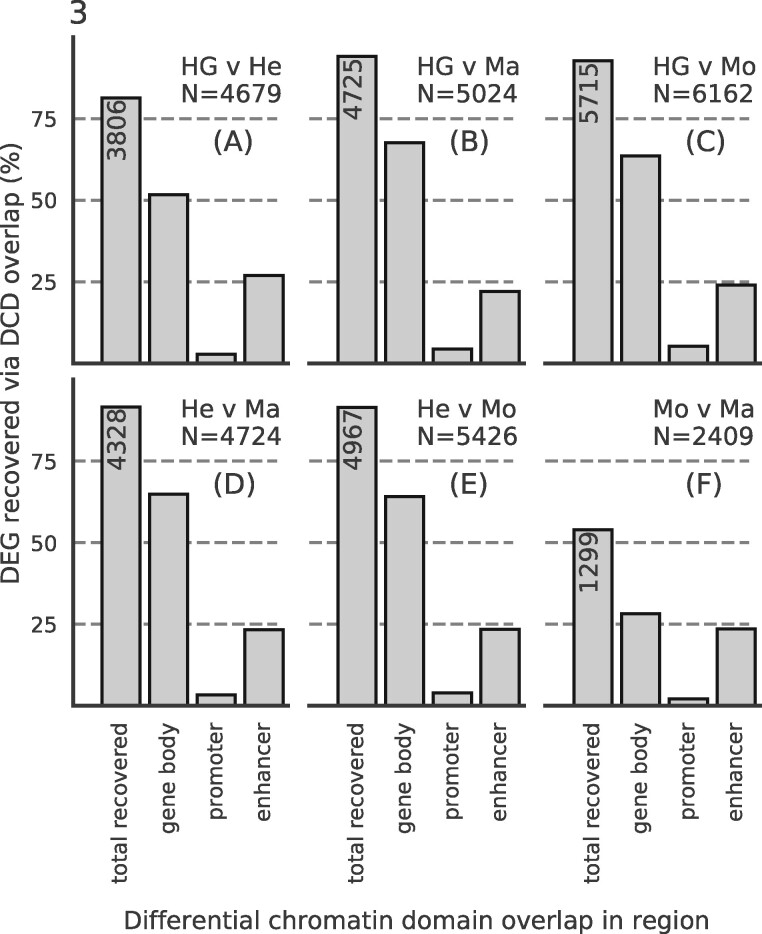
DCDs recover DEGs: bar heights indicate percentage of recovered DEGs by counting overlaps with DCDs in gene bodies, in gene promoters (but not in gene bodies) or in gene-associated enhancers (but not in gene bodies or gene promoters). The leftmost bar is annotated with the total number of recovered genes. *N*, total number of DEGs per comparison (**A**)–(**F**)

We investigated to what extent DCDs can be used to specifically identify differentially expressed genes (DEGs). As ground truth for this analysis, we used the same DESeq2 results as in the previous section, but applied a threshold to split the genes into differentially expressed and stable ones (see Supplementary Methods). First, we checked what percentage of DEGs could be recovered using SCIDDO’s DCDs (Fig. 3). For four out of the six sample comparisons, more than 90% of all DEGs could be recovered with DCDs either overlapping the gene body, the gene promoter or at least one gene-associated enhancer. For the comparison of HepG2 to primary hepatocytes (Fig. 3A), ∼81% of DEGs could be recovered, and for the comparison of monocytes to macrophages, 54% of all DEGs were recoverable by using DCDs (Fig. 3F). The comparatively lower rate of DEG recovery for the monocyte to macrophage comparison seems to be in line with the already observed trend of fewer differences on the chromatin level with increasing cellular relatedness (e.g. see Fig. 2). We present a more in-depth analysis of this observation as part of Supplementary Result S2.5. Notably, the seemingly low number of promoters with DCD overlap results from counting each DCD only once, i.e. a DCD overlapping the gene body and reaching into the promoter region is only counted as overlapping a gene body (see Supplementary Fig. S10 for the same analysis but allowing for multiple counts per DCD).

Next, we tested if it was possible to broadly distinguish between DEGs and stably expressed genes by thresholding on the *E*-values of the DCDs that overlap gene bodies. To that end, we stratified the set of DEGs based on their fold change into three groups (top 20%, middle and bottom 40%) and plotted the *E*-value distribution of the DCDs for these three groups and for all other chromatin domains (Supplementary Fig. S11, bottom panel). We find that DEGs with the highest fold change in expression overlap DCDs that have a significantly higher *E*-value on average relative to DCDs overlapping the remaining DEGs. Furthermore, it can be observed that the *E*-value distribution of the DCDs overlapping stable genes is similar to those that do not overlap any gene (but could, e.g. overlap with intergenic enhancers). The number of distinct DCDs that overlap any given gene shows no notable variation across all groups (Supplementary Fig. S12, middle panel). The distribution of the gene body lengths in the respective groups appears to be fairly balanced (Supplementary Fig. S12, top panel) and thus does not suggest that the number of DCD overlaps or the observed difference in *E*-value distribution is an effect of gene body length. We explicitly confirmed this by repeating the analysis, but this time stratifying DEGs by gene body length (Supplementary Fig. S12). The *E*-values of the DCDs overlapping the longest genes are comparatively lower, and this suggests that larger *E*-values are probably not a result of increasing gene body length.

### 3.5 SCIDDO affords direct interrogation of chromatin dynamics

**Fig. 4.**
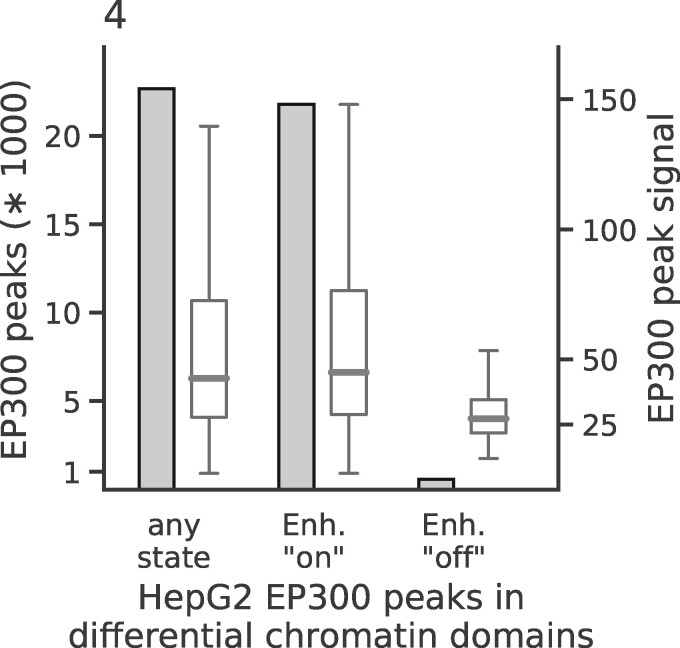
Chromatin dynamics at HepG2 enhancer elements: height of the bars depicts total number of peaks overlapping DCDs (left *y*-axis) and box plots show distribution of the signal of the overlapping EP300 peaks (right *y*-axis). The three groups represent EP300 peaks overlapping with DCDs in general (left); with DCDs restricted to genomic locations showing an enhancer ‘on’ state in HepG2 (middle); with DCDs restricted to genomic locations showing an enhancer ‘off’ state in HepG2. For all three groups, the DCDs identified in the HepG2 to monocyte comparison were used

A noteworthy feature of SCIDDO is the possibility to filter DCDs by chromatin dynamics. Given that chromatin states generated by the CMM18 model have been assigned meaningful labels (Supplementary Fig. S1 and Table S3), users can exploit this easily interpretable annotation to filter DCDs. We used this feature in combination with external validation data to investigate if it is possible to identify enhancers that switch from an ‘on’ to an ‘off’ state between two cell types. To this end, we selected two sets of chromatin state labels as representing active and inactive enhancer states (see Supplementary Methods). SCIDDO then uses these state labels to filter the DCDs and, by default, returns those subregions of a DCD where the chromatin change of interest can be observed between the selected cell types. It should be emphasized that, while the chromatin dynamics filtering is based on the identified DCDs, the individual subregions returned by SCIDDO cannot be statistically evaluated by computing an *E*-value. Subregions of a DCD can be as short as one or two genomic bins and, thus, the computed *E*-value of a subregion is unlikely to indicate statistical significance. For comparison, we downloaded several ENCODE peak datasets of the transcriptional co-activator EP300 (p300) for the cell line HepG2 (see Section 2 and Supplementary Methods). Though EP300 is known to be highly predictive of tissue-specific enhancer activity (Visel *et al.*, 2009), it cannot be assumed that all downloaded EP300 peaks mark active enhancers that are unique to HepG2, and are hence inactive in any other cell type. As a consequence, an exhaustive overlap between EP300 peaks and (switching) enhancer regions in DCDs cannot be expected. Instead, we hypothesized that it is more realistic to assume that any biologically meaningful enhancer switch within a DCD subregion should likely also show a change in EP300 occupancy. We investigated this hypothesis by plotting the count of EP300 peaks and their signal strength for all peaks generally overlapping DCDs, and for all peaks overlapping with DCD subregions showing enhancer switches from ‘on’ to ‘off’ and vice versa from ‘off’ to ‘on’ for the comparison of HepG2 to monocytes (Fig. 4). There is a prominent difference both in absolute number of peaks and in signal strength for the two directions of enhancer switching. This example illustrates that SCIDDO can also offer support in downstream analysis by quickly identifying regions of specific and directed changes on the chromatin level.

### 3.6 DCDs recover DEGs with increased stability compared to individual histone marks

The number of available tools that use chromatin state maps as input for a differential analysis is limited. ChromDet (Carrillo-de Santa-Pau *et al.*, 2017) is designed for group comparisons with at least 5–10 replicates each (*personal communication*), and thus did not give results on our dataset. Similarly, ChromDiff (Yen and Kellis, 2015) could not identify any differential chromatin marking (in genes), presumably due to lacking statistical power given the limited number of replicates in our dataset. The Chromswitch package (Jessa and Kleinman, 2018) can only process one chromatin state at a time, which complicates direct and fair comparisons with the DCDs identified by SCIDDO.

**Fig. 5.**
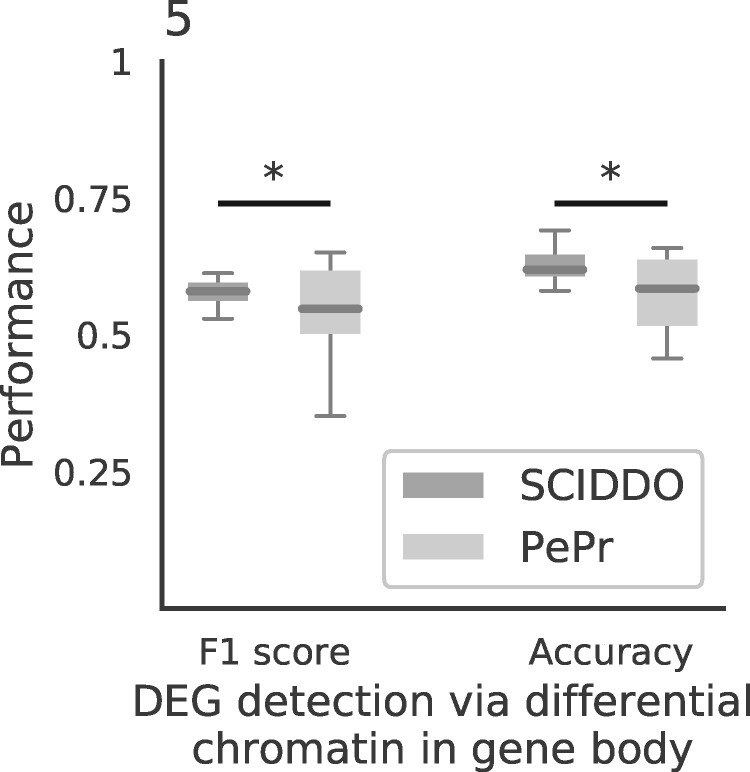
SCIDDO shows more stable performance at detecting DEGs: box plots depict SCIDDO’s and PePr’s (light grey) performance of detecting DEGs quantified as *F*1 score (left) and as accuracy (right). Performance values are summarized over all sample group comparisons and for different thresholds on gene expression fold change (0.5, 1, 2 and 4) and on adjusted *P*-values (0.1, 0.05, 0.01 and 0.001) computed with DESeq2 to call DEGs. At least one DCD/differential H3K36me3 peak (PePr) was required in the gene body of a DEG to be considered detected on the chromatin level. Differences in performance were assessed with a one-sided Mann–Whitney U test and considered significant ‘*’ at *P* < 0.01

We thus decided to compare SCIDDO to PePr (Zhang *et al.*, 2014), an established tool for the differential analysis of individual histone marks that can process replicated samples. This strategy has the advantage of reflecting the canonical ‘rule-based’ approach for interpreting histone marks in well-characterized regulatory contexts, e.g. by determining enhancer activity based on the presence of H3K27ac peaks (Creyghton *et al.*, 2010). Specifically, we used PePr to perform a differential analysis for the same six sample group comparisons and evaluated PePr’s and SCIDDO’s performance for the task of detecting DEGs based on differential chromatin marking. To this end, we considered two different scenarios: first, genes overlapping with at least one DCD (SCIDDO) or having at least one H3K36me3 peak in one cell type but none in the other cell type (PePr) were labeled as differentially expressed. This strategy could be applied to all 20 091 genes in our gene annotation (gene set G1). In the second scenario, differential chromatin in gene bodies was taken into account in the same way, but as an additional requirement, at least three annotated enhancers of a gene had to show differential chromatin marking (H3K27ac peaks for PePr) to label the gene as differentially expressed. This reduced the number of genes in the evaluation set to 17 735 (88.3%; gene set G2), i.e. all genes that had at least three enhancers annotated. We compared the chromatin-based labeling of genes in sets G1 and G2 with the ground truth computed with DESeq2 (Love *et al.*, 2014). While we settled for a fix threshold on gene expression fold change (>2) and *P*-value (<0.01) to identify DEGs throughout this study, we varied these values for the comparison between SCIDDO and PePr to examine the stability of their performance for different levels of differential expression stringency. We calculated accuracy and *F*1 score for all sample comparisons and the gene expression fold changes 0.5, 1, 2 and 4 and *P*-values 0.1, 0.05, 0.01 and 0.001 for the two gene sets G1 and G2 (Fig. 5 and Supplementary Fig. S17). In summary, SCIDDO’s performance is superior to PePr. Averaged over all comparisons, SCIDDO shows an accuracy of 64.6% (G1) and 69.2% (G2) and an *F*1 score of 57.5% (G1) and 59.1% (G2) for the two different strategies of labeling a gene as differentially expressed. For PePr, the average performance scores are 57.6% (G1) and 57.7% (G2) accuracy and 54.6% (G1) and 54.7% (G2) *F*1 score.

Since PePr has no notion of a ‘quiescent’ chromatin state, we repeated the above comparison without treating the quiescent chromatin state as ‘not differential’ by default in the SCIDDO analysis (see Supplementary Methods). In this scenario, SCIDDO’s performance slightly increased (Supplementary Figs S18G1 and S19G2) at the expense of decreased interpretability for DCDs with a chromatin state composition largely dominated by the quiescent state.

## 4 Discussion

The use of chromatin state segmentation maps for large-scale annotation and interpretation of reference epigenomes is well established in the field of computational epigenomics [see, e.g. Ernst *et al.* (2011) and Ernst and Kellis (2015)]. Nevertheless, comparatively little effort has been invested in the development of generally applicable software that assists researchers in exploiting these resources. To fill that gap, we developed SCIDDO, a new tool that implements a score-based approach for the fast detection of DCDs between potentially small groups of replicated samples.

The results presented above indicate that SCIDDO’s score-based approach is able to robustly identify consistent sets of differential chromatin candidate regions across individual biological replicate comparisons. This observation suggests that SCIDDO is well-equipped for the commonly encountered situation of limited replicate availability while still offering a statistically sound evaluation of the detected DCDs. Though the statistics implemented in SCIDDO do not afford a theory-driven evaluation of the detected DCDs, e.g. no suitable *E*-value threshold is motivated by the theory, we could validate our findings in several biologically meaningful ways. The considerable overlap between the detected DCDs and various regulatory annotation datasets (Fig. 2) suggests a functional role for the identified DCDs that is in line with published studies (Barski *et al.*, 2007; Heintzman *et al.*, 2007; Mikkelsen *et al.*, 2007). By relating gene expression fold changes to DCD formation in gene bodies and gene-associated enhancers, we could show that this presumed functional role seems indeed to have a measurable effect on gene expression behavior (Supplementary Figs S7–S9). Our findings conform to the established view that extensive chromatin changes in gene bodies as well as in gene-associated enhancers are suitable indicators of the expected gene expression fold change (Karlić *et al.*, 2010; Kouzarides, 2007; Plank and Dean, 2014). It should be emphasized that SCIDDO realizes this view on the interplay between chromatin changes and altered gene expression without directly quantifying differences on, e.g. the read count level. Nevertheless, SCIDDO is able to detect most DEGs (Fig. 3) and shows a performance in such tasks that is on average superior and more stable compared to competing approaches which implement much more time-intensive strategies to differential chromatin analysis (Fig. 5 and Supplementary Figs S17–S19). Taken together, the evidence supports the conclusion that SCIDDO’s score-based approach to differential chromatin analysis discovers biologically meaningful and interpretable DCDs.

An observable trend in the dataset we analyzed is the more limited variation on the chromatin level with increasing cellular relatedness, e.g. what we have detailed for the monocyte to macrophage comparison (Supplementary Result S2.5). While this inverse relationship is plausible, it implies that there is a natural limit in ‘resolution’ of differential chromatin state analyses that governs SCIDDO’s applicability in discerning cellular phenotypes, or in characterizing differentiation pathways. Although we did not investigate these potential limitations in depth, we collected multiple lines of evidence that illustrate various ways of how gene expression changes, and thus different cellular phenotypes, could be realized without necessarily leaving a detectable trace on the chromatin level (Supplementary Result S2.5 and Fig. S15). One of these blind spots in chromatin state maps is the ‘quiescent’ background state, i.e. the chromatin state without any detectable signal. If possible, a more fine-grained characterization of the background state would be a promising way of extending score-based differential chromatin analyses to cover even more regions of the (epi-) genome. To give an example, a widespread background state in gene bodies in only one sample group might be interpreted as biologically meaningful (Supplementary Result S2.5 and Fig. S14), and thus, an adapted scoring for the background state in this context could plausibly increase DEG recovery rates via DCD overlap.

Adaptations to the pairwise chromatin state scoring could be realized in a multitude of ways in future studies. While our approach based on the JSD has the benefit of not being affected by biases in our dataset, which might be an issue for data-derived scoring systems, it is also not customized to any particular notion of differential chromatin. It is one of SCIDDO’s distinguishing features that the user can specify any scoring scheme that fulfills the statistical assumptions and use for differential chromatin analysis. It is thus conceivable to study only a specific repertoire of dynamic chromatin changes given an appropriately chosen scoring matrix, e.g. focusing on enhancers and ignoring transcribed regions. Apart from such specific objectives, it would also be intriguing to investigate if, for a given state segmentation model, generally applicable scoring systems could be derived that are sensitive to the degree of cellular relatedness. In analogy to genome sequence analysis (Pearson, 2013), this could provide a different view on the dynamic epigenome in the course of cellular development.

## Supplementary Material

btaa960_Supplementary_Data
