# Supplementary material for: Fast detection of differential chromatin domains with SCIDDO
Source: Bioinformatics. 2020 Nov 24;37(9):1198–205. doi: 10.1093/bioinformatics/btaa960 (PMC8189691; doi:10.1093/bioinformatics/btaa960)
Supplement: btaa960_Supplementary_Data [file btaa960_supplementary_data.pdf]

Supplementary Material

Fast Detection of Differential Chromatin Domains with SCIDDO

Peter Ebert and Marcel H. Schulz*

*To whom correspondence should be addressed:
Cluster of Excellence on Multimodal Computing and Interaction, Saarland Informatics Campus, Germany. E-mail: mschulz@mmci.uni-saarland.de

Contents

1 Supplementary Methods 3
- 1.1 Overview of experimental data . . . 3
- 1.2 Generation of chromatin state maps . . . 3
- 1.3 Differential gene expression analysis . . . 4
- 1.4 Differential histone peak calling . . . 4
- 1.5 Chromatin dynamics at EP300 peaks . . . 4
- 1.6 Generation of randomly sampled genomic regions . . . 4
- 1.7 Algorithm 1: computation of length normalization factor L . . . 5
- 1.8 Fit of random scores to Gumbel-type extreme value distribution . . . 6

2 Supplementary Results 7
- 2.1 Differential chromatin scores follow extreme value distribution . . . 7
- 2.2 SCIDDO robustly identifies differential chromatin domains . . . 7
- 2.3 Chromatin state transitions in DCDs show consistent patterns of changes in genomic activity . . . 8
- 2.4 DCDs overlapping regulatory regions show higher E-values . . . 8
- 2.5 Methodological and biological limitations for chromatin-based detection of differentially expressed genes . . . 9

3 Supplementary Figures 12

4 Supplementary Tables 30

5 Supplementary References 34

List of Figures

S1 Chromatin state mnemonics and colors . . . 12
S2 Observed maximal scores and parameter estimates follow theoretical assumptions . . . 13
S3 Candidate regions are robustly identified across individual replicates . . . 14
S4 Chromatin state transitions in DCDs . . . 15
S5 DCD length distribution . . . 16
S6 E-value distribution in regulatory regions . . . 17
S7 DCDs affect gene expression (HG vs He / He vs Ma . . . 18
S8 DCDs affect gene expression (HG vs Ma / Mo vs Ma) . . . 19
S9 DCDs affect gene expression (He vs Mo / HG vs Mo) . . . 20
S10 DCDs recover DEGs . . . 21
S11 E-value distribution in DEGs by gene expression change . . . 22
S12 E-value distribution in DEGs by gene body length . . . 23
S13 Relaxing E-value threshold does not improve detection of short DEGs . . . 24
S14 Limitations for DEG recovery via DCDs . . . 25
S15 Difference in annotated miRNA targets and 3p UTR length . . . 26
S16 SCIDDO shows more stable performance at detecting DEGs . . . 27
S17 SCIDDO shows more stable performance at detecting DEGs . . . 28
S18 SCIDDO shows more stable performance at detecting DEGs . . . 29

List of Tables

S1 Histone data overview . . . 30
S2 Expression data overview . . . 31
S3 CMM18 state descriptions . . . 32
S4 SCIDDO runtime . . . 32
S5 E-value correlation in replicate comparisons . . . 33

1 Supplementary Methods

1.1 Overview of experimental data

All analyses were carried out using the official IHEC human hg38/GRCh38 assembly available at www.epigenomes.ca/data/CEMT/resources. We selected the following high quality DEEP samples to include both closely related as well as more distantly related cell types in our analysis: two replicates of HepG2 (HG 1 and 2; Supplementary Table S1), two replicates of hepatocytes (He 2 and 3; Supplementary Table S1), three replicates of monocytes (Mo 1, 3, and 5 [Wallner et al., 2016]) and two replicates of macrophages (Ma 3 and 5 [Wallner et al., 2016]). All primary cell types were isolated from healthy, adult donors. For each replicate, we downloaded the DEEP reference alignments for six histone marks (H3K4me1, H3K4me3, H3K27ac, H3K27me3, H3K36me3, H3K9me3) and the corresponding Input control as BAM files (Supplementary Table S1). Additionally, we downloaded DEEP mRNA expression data for all samples as raw read FASTQ files (Supplementary Table S2). The hg38 genome reference was restricted to fully assembled auto- and gonosomes for all data preprocessing steps. The differential analysis with SCIDDO was then limited to autosomes and chromosome X to alleviate any effects arising from the uneven distribution of sexes in our dataset. Annotation data were likewise limited to the same set of chromosomes. The GeneHancer [Fishilevich et al., 2017] enhancer annotation was licensed for academic use on 2017-05-30. The GeneHancer annotation was reduced to gene-enhancer pairs that could be mapped to gene identifiers in the GENCODE v21 annotation [Harrow et al., 2012]. The analysis of chromatin dynamics at enhancer regions was based on EP300 data downloaded from ENCODE [The ENCODE Project Consortium et al., 2012] under accessions ENCFF674QCU and ENCFF806JJS.

1.2 Generation of chromatin state maps

Following IHEC recommendations, all histone BAM files were filtered using Sambamba v0.6.6 [Tarasov et al., 2015] to exclude low quality reads (mapping quality ≥ 5; no duplicated, unmapped or non-primary reads/alignments). These filtered BAM files were used as input to generate chromatin state segmentation maps for all samples. We used the pre-trained 18-state ChromHMM (CMM18) model provided by the Roadmap Epigenomics Mapping Consortium (REMC [The Roadmap Epigenomics Consortium et al., 2015]). We decided to use this pre-trained model because it has been carefully designed using the large compendium of epigenomes generated by the REMC. We thus assumed that this model robustly captures chromatin states irrespective of the biological source of the samples at hand. As an additional benefit, the chromatin states of the CMM18 model were functionally characterized and labeled by the REMC to make interpretation of the state segmentation maps straightforward (Supplementary Figure S1 and Table S3). We executed version 1.12 of ChromHMM with commands `BinarizeBam -b 200` and `MakeSegmentation -b 200` and otherwise default parameters to create the state segmentation maps.

1.3 Differential gene expression analysis

Gene expression estimates per replicate were computed with Salmon v0.9.1 [Patro et al., 2017] using the GENCODE v21 [Harrow et al., 2012] annotation for protein coding genes. For each gene in the GENCODE reference, we extracted genomic coordinates for the gene body (5' to 3' end) and for the promoter (-2500 bp to +500 bp around the 5' end) using custom scripts (see section "Availability of raw data and code" for link to sources). After expression quantification, we used DESeq2 v1.18.1 [Love et al., 2014, Soneson et al., 2016] to obtain differential expression estimates for all six possible pairs of sample replicate groups in our dataset. We split the DESeq2 results into groups of differentially expressed genes (DEGs) and non-differentially expressed genes (stable genes) based on an absolute log2 fold change in expression of at least 2 and a multiple testing corrected p-value of less than 0.01.

1.4 Differential histone peak calling

We selected PePr [Zhang et al., 2014] as a current state-of-the-art tool for differential chromatin analysis as a reference to compare to. We executed PePr v1.1.18 to perform differential analysis including postprocessing for all six possible pairs of sample replicate groups in our dataset. All available replicates were processed in a single run of PePr for each comparison. PePr was executed with histone peak type set to `broad` for the mark H3K36me3, and otherwise default parameters. The resulting histone peak sets were filtered to peaks with a q-value of less than 0.01 using custom scripts (see section "Availability of raw data and code" in the main text for link to sources).

1.5 Chromatin dynamics at EP300 peaks

EP300 peak datasets for HepG2 were downloaded from ENCODE [The ENCODE Project Consortium et al., 2012] (ENCFF674QCU and ENCFF806JJS) and merged using bedtools v2.26.0 [Quinlan and Hall, 2010]. For the chromatin dynamics filtering, chromatin states 7–11 (genic, active and weak enhancers) were considered as enhancer "on" states, and chromatin states 13, and 15–17 (heterochromatin, bivalent enhancer and polycomb repression) were considered as enhancer "off" states.

1.6 Generation of randomly sampled genomic regions

For each set of DCDs, we generated randomly sampled genomic regions as control set. The DCDs were used as input to a custom script that generated a random sample of genomic regions following the DCD length distribution. Since this process was performed in parallel over chromosomes, the generated control regions resemble the DCDs both in length and in chromosomal distribution. Additionally, the control regions for a specific set of DCDs are disjoint (as DCDs are also disjoint by construction). To limit the runtime of the sampling process, a lower limit of at least 95% generated control regions was set (see section "Availability of raw data and code" in the main text for link to sources).

1.7 Algorithm 1: computation of length normalization factor L

The Expect (E) value of a differential chromatin domain is computed according to the formula

$$E = K \cdot L \cdot e^{-\lambda R} \tag{1}$$

where the value L is the length of the chromosomal sequence, R is the raw score of the segment, and K and λ are the Karlin-Altschul parameters that are estimated by external routines (see main text for references). The length normalization factor L is computed as follows:

Algorithm 1: Compute length normalization factor L

Input: chromatin state maps for 2 sample groups (GROUPS), each consisting of at least one replicate r

Output: Length normalization factor L_c per chromosome c

```
for c ∈ CHROMOSOMES do
    L_c = 0
    n = |c|
    for G ∈ GROUPS do
        states_{1...n} = ∅
        for r ∈ G do
            L_c = L_c + Σ_{p=1}^{n} 𝟙(r_p ∉ states_p)
            for p = 1 to n do
                states_p = states_p ∪ s_p
            end
        end
    end
    L_c = L_c − n
end
```

To give a simple and explicit example, consider the groups $G_1 = \{(A, B, B), (A, B, C), (A, B, A)\}$ and $G_2 = \{(A, B, C), (A, B, C)\}$. Running Algorithm 1 on this dataset would result in the following summation: $G_1 : 3+1+1$ and $G_2 : 3+0 \rightsquigarrow L_c = (3+1+1)+(3+0)-3 = 5$. Note that Algorithm 1 is used when SCIDDO's parameter `--adjust-group-length` is set to `adaptive` for the `scan` subcommand. This is the recommended setting for comparing two groups of high-quality (replicated) samples. For cases where the sample groups to be compared are heterogeneous, the `--adjust-group-length` parameter should be set to `linear`. For the `linear` setting, the length normalization factor L_c will simply be computed as $L_c = |c| \cdot \#comparisons$. For the example above, the result would then be $L_c = 3 \cdot 6 = 18$.

1.8 Fit of random scores to Gumbel-type extreme value distribution

The calculation of the E-value (see Materials and Methods in the main text) assumes a null model of random sequences. Following the theory (cf. Theorem 1 in [Karlin et al., 1990] and examples in [Karlin and Altschul, 1993]) the normalized maximal scores should follow a Gumbel-type extreme value distribution when comparing random state sequences, in the limit of the sequence length n. Since SCIDDO supports the use of customized scoring schemes, it also supports the user in assessing if the chosen scoring scheme follows this theoretical assumption. To that end, SCIDDO scans the randomly shuffled chromatin state maps of all sample pairs for high scoring subsegments and retains only the maximally scoring subsegment per chromosome; if several segments with identical scores emerge, only the first one is kept. This process is iterated until a pre-specified number of these "random" scores have been found. The scores underlying Figure S2A have been generated in that way. The user can then use these "random" scores and, e.g., assess their fit to a Gumbel-type extreme value distribution following our example in Figure S2A. Notably, in Figure S2A, we jointly fitted all "random" scores of all chromosomes to simplify the visualization.

2 Supplementary Results

2.1 Differential chromatin scores follow extreme value distribution

The last step in the SCIDDO workflow described main Figure 1 consists of turning the differential chromatin scores (DCSs) into an E-value that is used for filtering the set of candidate regions to obtain the final set of differential chromatin domains (DCDs). This step relies on theory developed for biological sequence analysis (see main text Materials and Methods) and requires first a normalization of the raw cumulative DCSs to account for the fact that comparing longer chromosomal sequences increases the chances of observing higher cumulative DCSs. This normalization uses two estimated statistical parameters, λ and K. These parameters have no biological interpretation, but can be thought of as scaling factors for the scoring system and the sequence length, respectively. Moreover, the theory assumes a null model of random sequences, and under this null model, the distribution of the scores should in the limit converge in distribution to a Gumbel-type extreme value distribution (see main text Materials and Methods). We confirmed that this is indeed the case in our analysis by comparing randomized chromatin state maps with each other and fitting all maximal DCSs identified during this sampling procedure to a Gumbel distribution (Figure S2A). We also plotted the per-chromosome estimates of the statistical parameters λ and K that are needed for the score normalization (Figure S2B; see Methods), and could confirm that the estimates are within reasonable bounds given examples from literature [Karlin and Altschul, 1993]. The observed agreement with theory thus supports the last step in the SCIDDO analysis (Main Figure 1 step (C)) of filtering candidate regions based on their E-value.

2.2 SCIDDO robustly identifies differential chromatin domains

Histone ChIP-seq data is known to be affected by various sources of noise, e.g., ranging from artifacts introduced during library preparation, to irregularities caused by varying mappability in the reference genome or to spurious signal due to unspecific antibody binding [Bailey et al., 2013, Head et al., 2014, Landt et al., 2012]. In combination, biological and technical variation can render any differential analysis pointless if the results are dominated by noise, and not by the biological signal of interest. To test if the identified candidate regions were indeed representative and not replicate-specific, we computed the Spearman correlation of the E-values between all overlapping candidate regions. We visualized an exemplary case selected based on the mean of all comparisons. This exemplary case shows a Spearman correlation of 0.72 between the candidate regions (Figure S3). The red bars in the lower left corner indicate candidate regions that are unique to the respective replicate comparison. It can be observed that unique candidate regions tend to have comparatively lower E-values whereas those candidate regions found in both replicate comparisons tend to have higher E-values. In general, the average Spearman correlations across all replicate comparisons are consistently in high range from 0.67 (HepG2 vs. hepatocytes) to 0.73 (HepG2 vs. monocytes; Table S5).

2.3 Chromatin state transitions in DCDs show consistent patterns of changes in genomic activity

For all six sample comparisons, we plotted the chromatin state transitions observed in the identified DCDs to examine if the overall change in chromatin activity showed comparison-specific characteristics (Figure S4). The chromatin state transition frequencies indicate a general trend that activity states related to transcription and transcriptional regulation are down-regulated by, e.g., polycomb-mediated silencing. For the four comparisons involving one liver and one blood cell type (Figure S4 B–E), a switch from polycomb-repressed to heterochromatin can also be observed quite frequently, indicating long-term silencing of the respective region. The state transitions for the monocytes versus macrophages comparison seem to show a different overall pattern, with more state switches within the borad functional categories related to (active) transcription and transcriptional regulation. In other words, when comparing more distantly related cell types, the identified DCDs seem to indicate rather broad changes in genomic activity including long-term silencing via heterochromatin formation. For the two closely related cell types monocytes and macrophages, the chromatin state switches appear to be more constrained to the respective functional activity level.

2.4 DCDs overlapping regulatory regions show higher E-values

The results presented in main Section 3.2 ("Differential chromatin domains occur in variousregulatory contexts") illustrate that the distribution of overlaps seems not to be affected by the number of DCDs identified. In all comparisons (main Figure 2), at least ~70% of the DCDs overlap with at least one regulatory region annotated in the Ensembl Regulatory Build [Zerbino et al., 2015]. The Regulatory Build comprises several different types of regulatory regions and has extensive genome coverage. Hence, the Regulatory Build enables us to interpret the relevance of DCDs in light of various functional categories. Since the distribution of genomic locations of the DCDs seems fairly similar across all comparisons, and analogous observations can be made when examining the length distribution of the DCDs (Supplementary Figure S5), we examined if there is a difference in DCD E-values aggregated over all comparisons (Supplementary Figure S6). DCDs overlapping any regulatory region show higher E-values compared to those DCDs that have no overlaps (Supplementary Figure S6, bottom panel). This effect is most pronounced for annotated promoters and transcription factor binding sites (TFBS), and this seems not to be an effect of regulatory region size (Figure S6, top panel). The average number of distinct regulatory region overlaps per DCD shows that a DCD often spans several of the shorter regulatory regions, with the exception of TFBS, which is the least abundant region type with a median size < 1 kbp in the Regulatory Build. At the other end of the size spectrum are promoters, which also show hardly any variation around a median of one DCD overlap per promoter.

2.5 Methodological and biological limitations for chromatin-based detection of differentially expressed genes

The theory borrowed from local scoring and implemented in SCIDDO is used to assign a measure of statistical stringency — the E-value — to each discovered DCD (see main Materials and Methods). Yet, the theory does not offer a way to decide what threshold on the E-value best separates genuine from chance observations. The necessary normalization to account for the length of the sequences being compared immediately suggests that short but biologically *true* differential regions will be assigned an (untransformed) E-value above SCIDDO's default threshold of 1.
We checked the extent to which the default E-value threshold of 1 could limit SCIDDO's ability to identify — especially short — DEGs. We binned all DEGs by their gene body size and plotted the amount of genes with a DCD overlapping their gene body at E-value thresholds of 1 and 100 (Supplementary Figure S13). The histogram shows the expected behavior of SCIDDO to predominantly recover longer DEGs by means of finding a DCD in their gene body. However, relaxing the E-value threshold seems not to affect this general trend as the additional DEGs also show a tendency toward longer gene bodies. We thus wondered if other technical or biological artifacts might exacerbate the detection of DEGs on the chromatin level. We focused specifically on the comparison of monocytes to macrophages where approximately only 54% of all DEGs could be recovered using DCDs (see main Figure 3F).

As a first step, we examined if artifacts in the data could be the reason for the low DEG recovery rate. Besides chromatin states with annotated function, chromatin state maps usually include a so-called background state that represents regions of no detectable signal (state number 18 labeled as "quiescent" in the CMM18 model). It is important to realize, though, that the interpretation of this background state is difficult. While it is conceivable that technical problems caused this lack of a signal in certain regions of the genome, it may be biologically meaningful in others. Moreover, the six canonical histone marks included in this study certainly cover a wide range of functionally important chromatin signals, but they do not represent the entire regulatory chromatin landscape. To give an example, the recently characterized H3K122ac histone modification is also found at active enhancers that lack the canonical H3K27ac marking [Pradeepa, 2017]. Given these uncertainties, we opted for a conservative approach and considered the background state as not differential relative to all other chromatin states (see main Materials and Methods).
We evaluated how many DEGs might not be recoverable under these conditions for the monocyte to macrophage comparison. For each of the 1110 DEGs that could not be recovered, we computed the percentage of the gene body length covered with the background state (averaged over all replicates in the respective groups). We found that close to a hundred genes that are covered to at least 60% with the background state are shared between the monocyte and the macrophage group (Supplementary Figure S14A). At a higher threshold of 80% body coverage, this number drops to 35 genes. Given that this considers genes that are in the same uninformative chromatin state to roughly the

same extent in all samples — and being differentially expressed at the same time — it seems justifiable to assume that the non-detection of these genes is not a limitation of SCIDDO. When focusing on the genes that are covered with the background state in either monocytes or macrophages, the numbers rise considerably (Supplementary Figure S14B). 164 genes are above the lower threshold of at least 60% coverage, and when raising the threshold to at least 80% coverage, 72 genes are still affected. In this scenario, the non-detection of the DEGs is hence largely driven by the lack of a signal in one of the two sample groups.

Considerations involving the background state might explain a few hundred cases of DEGs that could not be recovered by SCIDDO. It follows that a considerable amount of genes were assigned biologically meaningful chromatin states and yet were not detectable.
We hypothesized that a plausible cause for this could be a comparatively weak change in gene expression for non-detectable genes. When a gene is switched from “off” to “on”, a substantial change in the histone marking can be expected. However, if the gene is already actively transcribed and then simply upregulated, e.g., by activating additional enhancer elements (cf. Supplementary Figures S7– S9), it is not obvious why this change in expression should lead to differential chromatin marking in the gene body. We tested this hypothesis by plotting the mean difference in expression, plus the minimal and maximal expression level in any sample, for all DEGs in the monocyte to macrophage comparison (Figure S14C–E). We split the genes into three groups based on DCD overlap in their gene body, in any associated enhancers but not in the body and no DCD overlap at all, i.e., the non-detectable genes. The mean change in gene expression is significantly higher in genes overlapping with a DCD compared to those genes that have no differential chromatin marking. Interestingly, the minimal expression level (Figure S14D) is still relatively high for those genes that show differential chromatin marking only in their enhancers. When relating the minimal to the maximal expression level (Figure S14D/E), the change in expression can be characterized as follows: genes with a DCD in their gene body jump from a low to a high expression level; genes with no DCD in their body but in their enhancer(s) show increased expression relative to an already high level, and genes with no DCD at all remain at a low to mildly elevated expression level. It should be pointed out that the implied directionality is supported by the observed expression changes for the monocyte to macrophage comparison (Supplementary Figure S8).

There is a multitude of mechanisms beyond the chromatin level that can fine-tune gene expression [Coulon et al., 2013, Kim and Ren, 2006, Orphanides and Reinberg, 2002]. Given that the DEGs lacking any sign of differential chromatin marking show also limited dynamics in their expression changes, we wondered whether there was any evidence of post-transcriptional control of these genes. As control group, we selected all genes that were not classified as differentially expressed but nevertheless showed signs of differential chromatin marking in their gene body (N=760 for the monocyte to macrophage comparison). We then plotted the number of annotated micro RNA targets

using the TargetScan v7.2 [Agarwal et al., 2015] annotation for both groups of genes (Supplementary Figure S15, bottom panel). There is indeed a small but statistically significant difference in the number of annotated micro RNA targets per gene between the two groups. This difference seems not to be caused by a difference in 3'-UTR length, where it is actually the group of DEGs without an overlapping DCD that has the larger 3'-UTR regions on average (Supplementary Figure S15, top panel).

3 Supplementary Figures

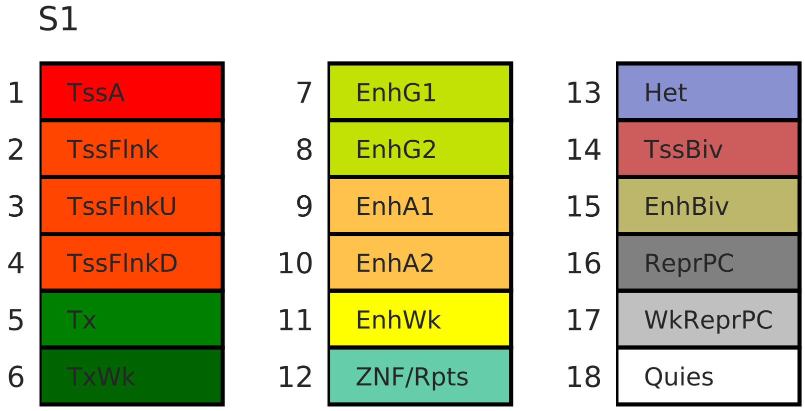

Figure S1: **CMM18 chromatin states**: mnemonics and colors for the 18 chromatin states of the ChromHMM CMM18 model provided by the REMC. See Table S3 for more detailed state descriptions.

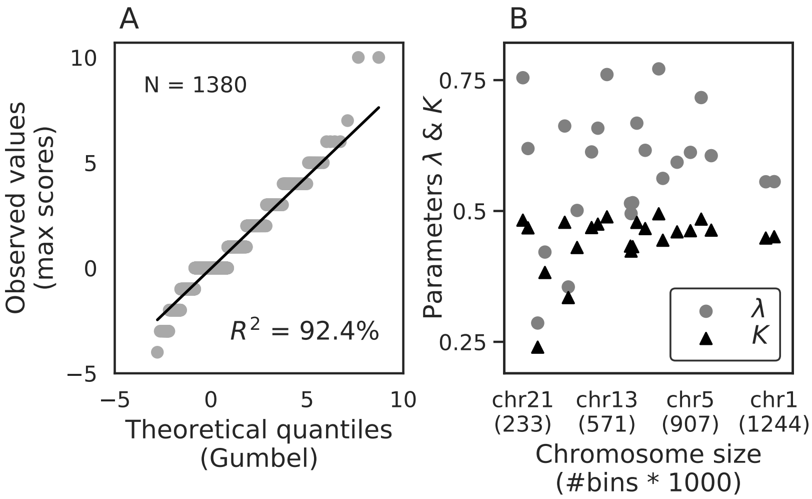

Figure S2: **Observed maximal scores and parameter estimates follow theoretical assumptions**: (A) Probability plot of all normalized maximal scores derived from comparing random sequences (y-axis) fit to the theoretical quantiles of a Gumbel–type extreme value distribution (x-axis). (B) Chromosomes are sorted by increasing size (in genomic bins) from left to right (x-axis) and the per-chromosome estimates of the two statistical parameters λ (gray points) and K (black triangles) are plotted on the same scale (y-axis). R^2: coefficient of determination

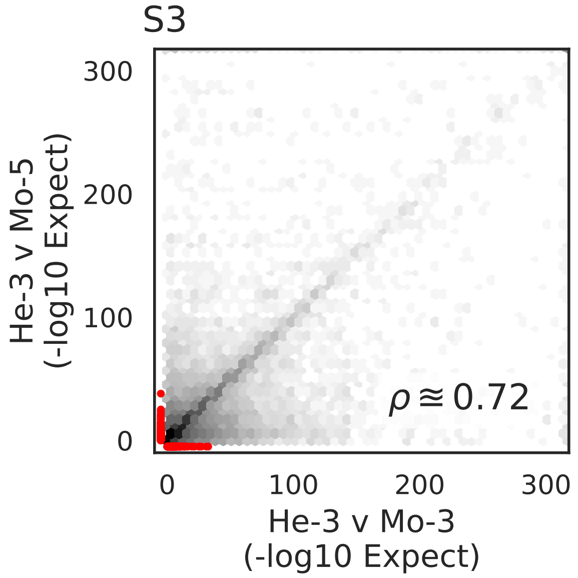

Figure S3: **Candidate regions are robustly identified across individual replicates**: exemplified agreement of candidate regions identified in replicate comparisons. E-values of candidate regions identified for He-3 vs. Mo-3 (x-axis) are plotted against E-values of overlapping candidate regions identified for He-3 vs. Mo-5 (y-axis). The red area indicates E-values of those candidate regions that are unique to the respective replicate comparison. ρ: Spearman correlation of E-values

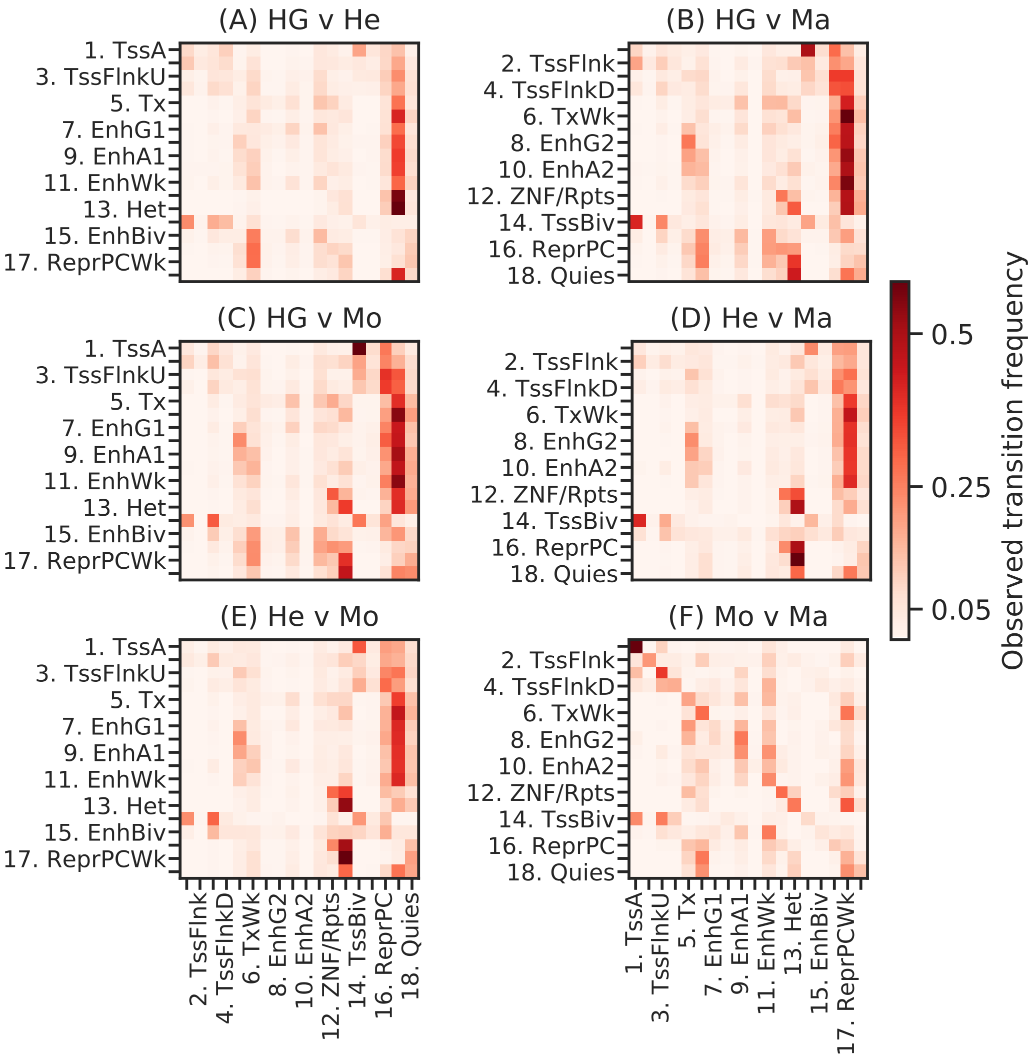

Figure S4: **Chromatin state transitions in DCDs**: frequency of chromatin state transitions observed in all DCDs for the respective sample comparison. State labels abbreviated as defined in Figure S1 and Table S3. Only half of the tick labels are shown for each heatmap to improve readability. Absolute counts of observed chromatin state transitions were turned into relative frequencies by dividing each row by the row total.

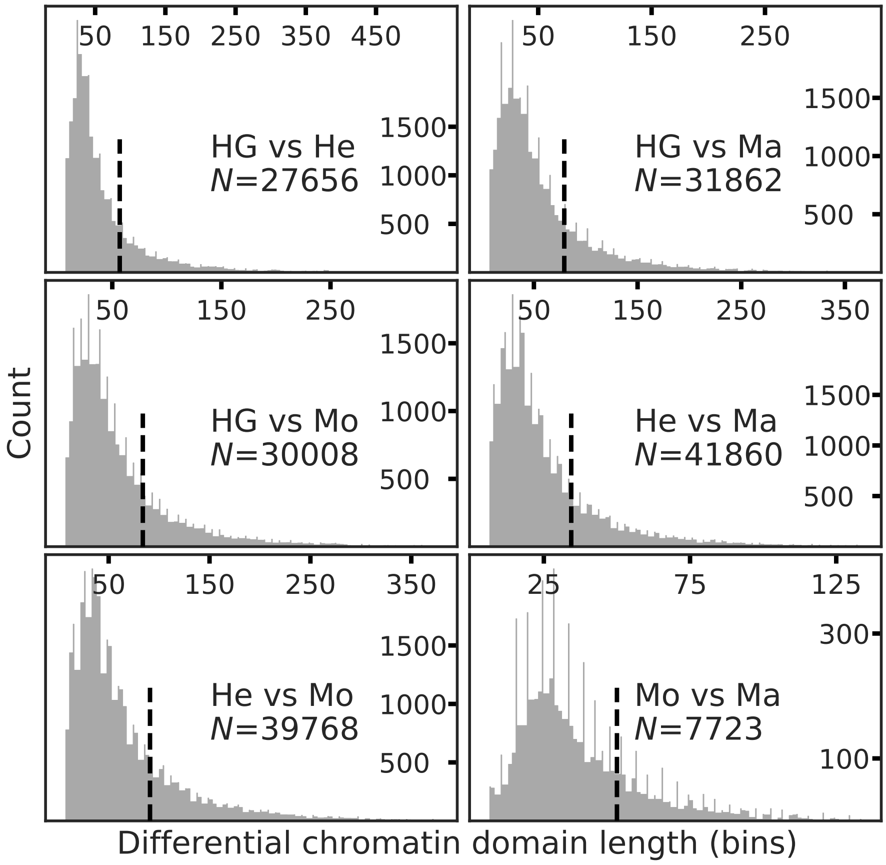

Figure S5: **DCD length distribution**: length distribution for the lower 97.5% (truncated for visualization) of all identified differential chromatin domains for the six sample group comparisons. The vertical line (dashed) marks the 75th percentile of the data. The DCD length is given in genomic bins à 200 bp (x–axis) N: total number of identified DCDs in the respective comparison.

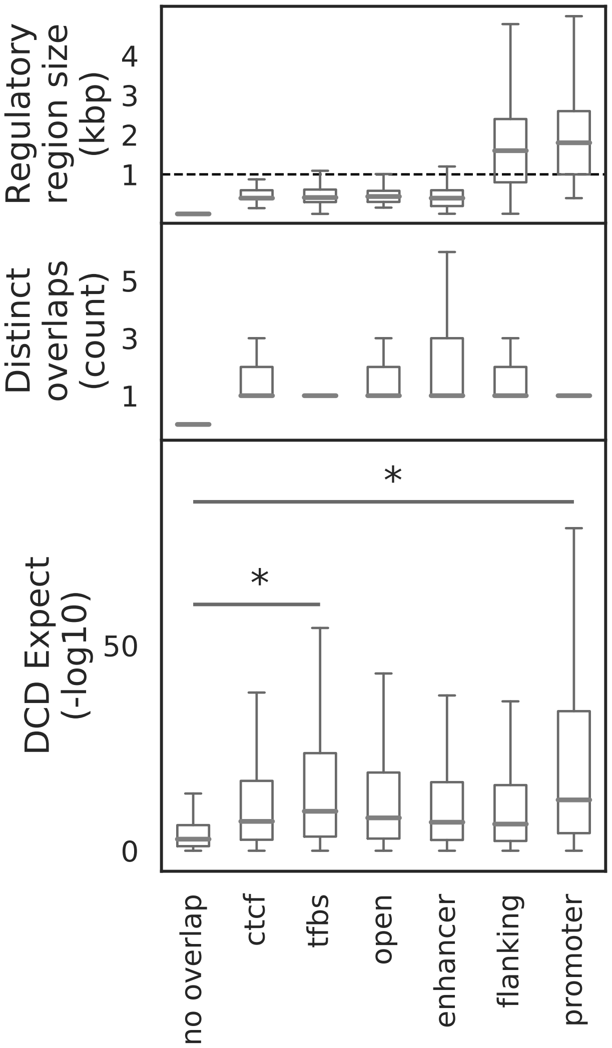

Figure S6: **E-value distribution of DCDs overlapping regulatory regions**: (bottom) box plots show distribution of E-values of all differential chromatin domains overlapping regulatory region types as annotated in the Ensembl Regulatory Build (v78) aggregated over all sample comparisons. Differences in magnitude of E-values were assessed with two-sided Mann-Whitney-U test and considered significant "*" at $p < 0.01$. (middle) box plots show distinct overlaps per DCDs, i.e., the number of regulatory regions of that type overlapping the same DCD. (top) size distribution of the Ensembl regulatory regions. Dashed line indicates a size of 1000 bp. Regulatory region types: ctcf: CTCF binding sites; tfbs: transcription factor binding sites; open: regions of open chromatin; enhancer: enhancer; flanking: promoter-flanking regions; promoter: promoter

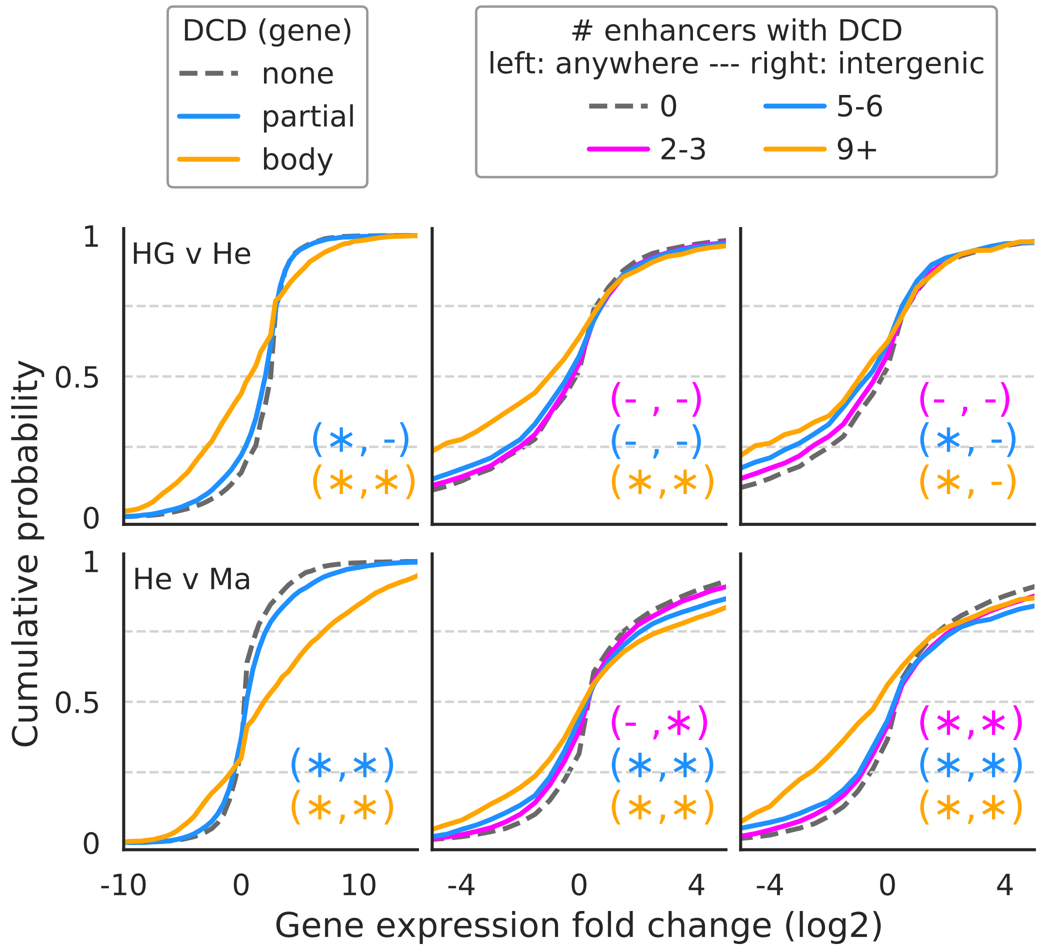

Figure S7: **DCD formation affects gene expression**: (left) all 20,091 genes were stratified by the amount of DCD overlap either covering more than 50% of the body (body; orange curve) or less than 50% of the body or the promoter region (partial; blue curve). Expression fold change of the genes in the respective groups is plotted along the x-axis within a restricted window for improved readability. Statistical significance of the difference in mean fold change of the groups relative to the no overlap group ("none") was computed separately for negative and positive fold change genes using a two-sided Mann-Whitney-U test ("*" significant with $p < 0.01$, "-" not significant otherwise). (middle/right) same analysis as for the gene body, but here counting the number of intra- and intergenic enhancers (anywhere, middle) or only intergenic enhancers (right) per gene that overlap a DCD. Expression fold changes plotted within a restricted window for improved readability. Statistical significance assessed as above.

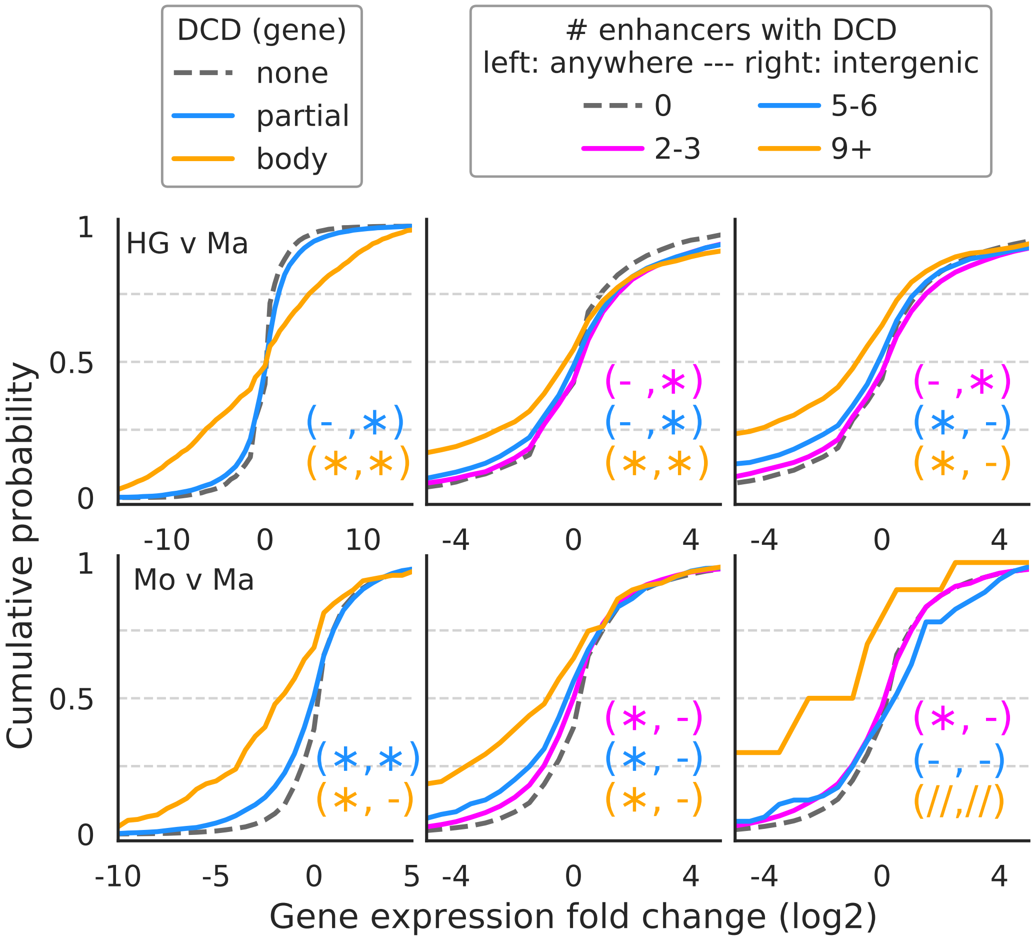

Figure S8: **DCD formation affects gene expression**: (left) all genes were stratified by the amount of DCD overlap either covering more than 50% of the body (body; orange curve) or less than 50% of the body or the promoter region (partial; blue curve). Expression fold change of the genes in the respective groups is plotted along the x-axis within a restricted window for improved readability. Statistical significance of the difference in mean fold change of the groups relative to the no overlap group ("none") was computed separately for negative and positive fold change genes using a two-sided Mann-Whitney-U test ("*" significant with $p < 0.01$, "-" not significant otherwise; "//": not enough data to compute test statistic). (middle/right) same analysis as for the gene body, but here counting the number of intra- and intergenic enhancers (anywhere, middle) or only intergenic enhancers (right) per gene that overlap a DCD. Expression fold changes plotted within a restricted window for improved readability. Statistical significance assessed as above.

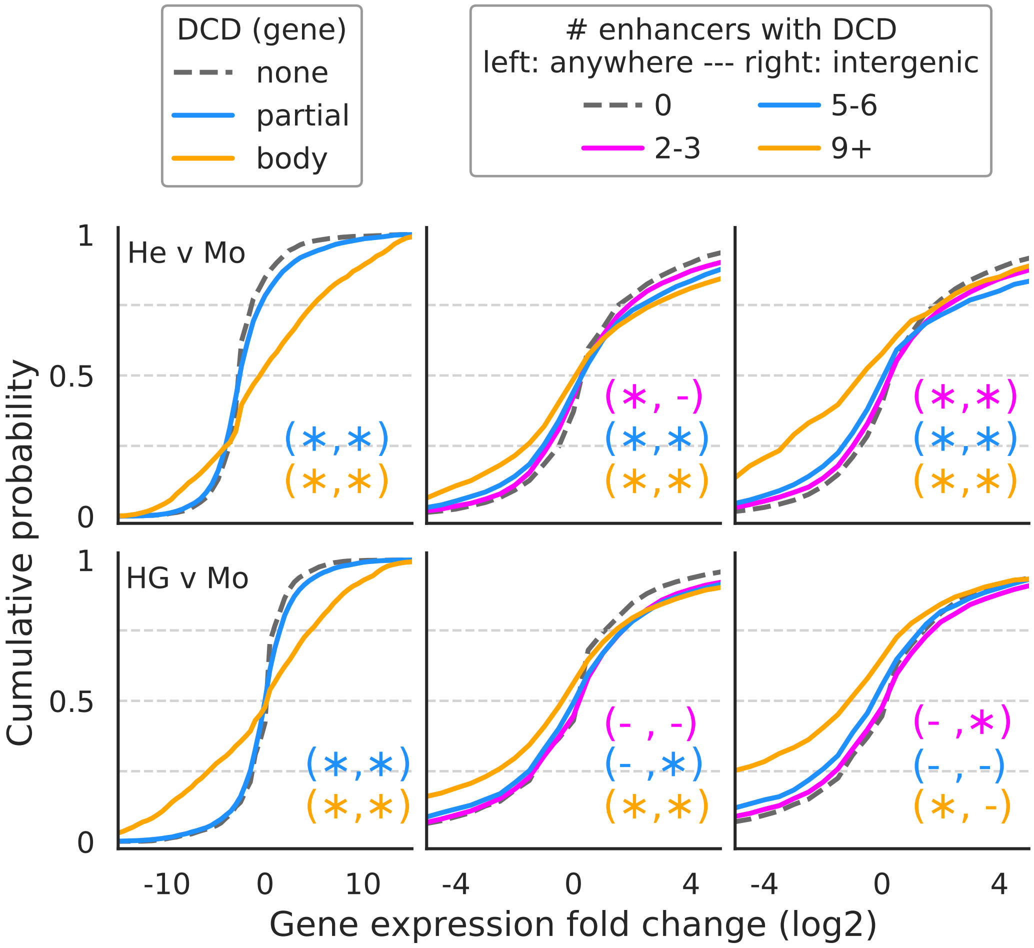

Figure S9: **DCD formation affects gene expression**: (left) all genes were stratified by the amount of DCD overlap either covering more than 50% of the body (body; orange curve) or less than 50% of the body or the promoter region (partial; blue curve). Expression fold change of the genes in the respective groups is plotted along the x-axis within a restricted window for improved readability. Statistical significance of the difference in mean fold change of the groups relative to the no overlap group ("none") was computed separately for negative and positive fold change genes using a two-sided Mann-Whitney-U test ("*" significant with $p < 0.01$, "-" not significant otherwise). (middle/right) same analysis as for the gene body, but here counting the number of intra- and intergenic enhancers (anywhere, middle) or only intergenic enhancers (right) per gene that overlap a DCD. Expression fold changes plotted within a restricted window for improved readability. Statistical significance assessed as above.

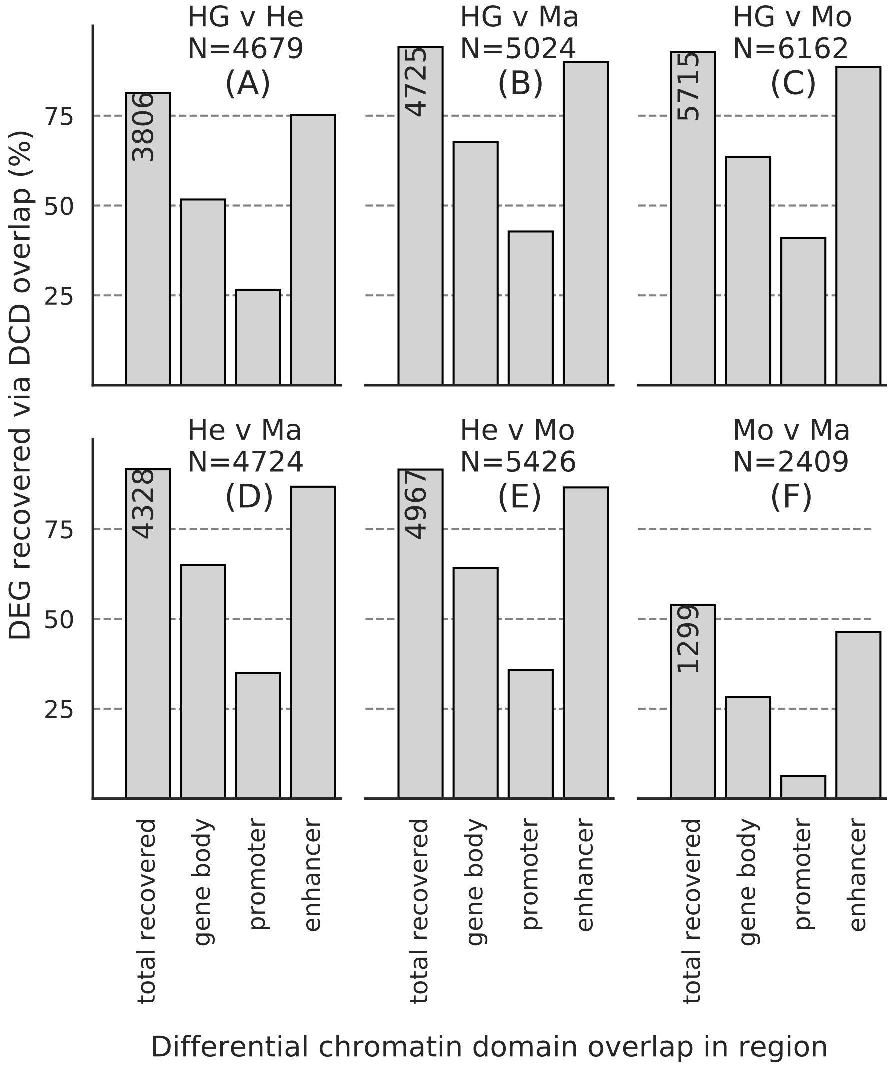

Figure S10: **Differential chromatin domains recover differentially expressed genes**: bar heights indicate percentage of recovered differentially expressed genes by counting overlaps with differential chromatin domains in gene bodies, in gene promoters or in gene-associated enhancers (i.e., this allows for multiple counts per DCD). The leftmost bar is annotated with the total number of recovered genes. N: total number of differentially expressed genes per comparison (A)–(F).

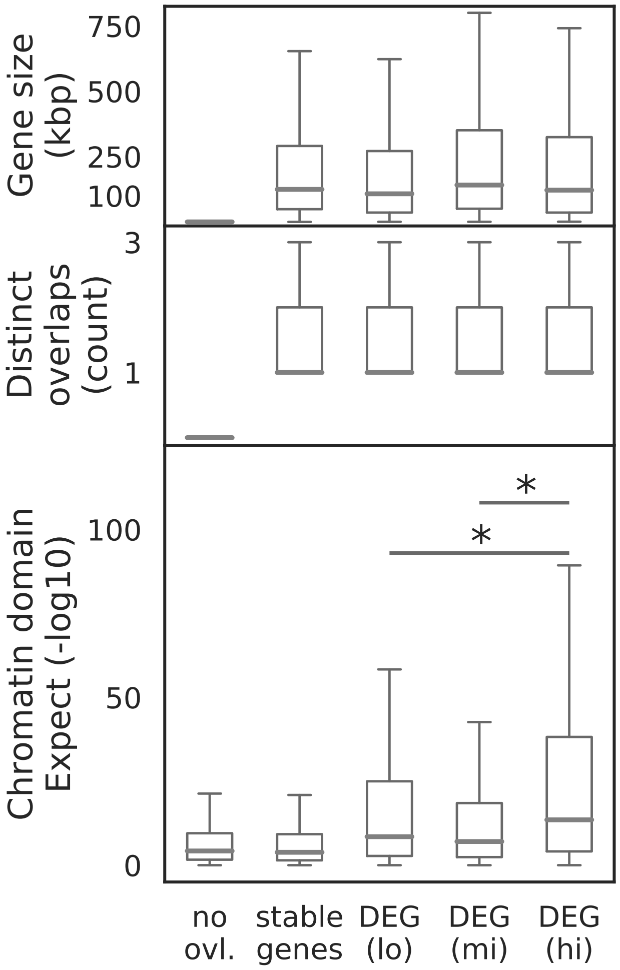

Figure S11: **E-value distribution in DEGs by gene expression change**: genes were stratified into four groups based on expression behavior (stable or differential) and the magnitude of expression change (lowest 40% [lo], middle 40% [mi] and highest 20% [hi] of DEGs according to gene expression fold-change). Bottom: boxplots show distribution of E-values of all DCDs overlapping gene bodies in the respective groups aggregated over all sample comparisons. The no overlap group contains all E-values of DCDs not overlapping any gene. Middle: boxplots show distinct DCD overlaps per gene. Top: boxplots show gene body length distribution of all genes in the respective group. Differences in magnitude of E-values were assessed with a two-sided Mann-Whitney-U test and considered significant (*) with $p < 0.01$.

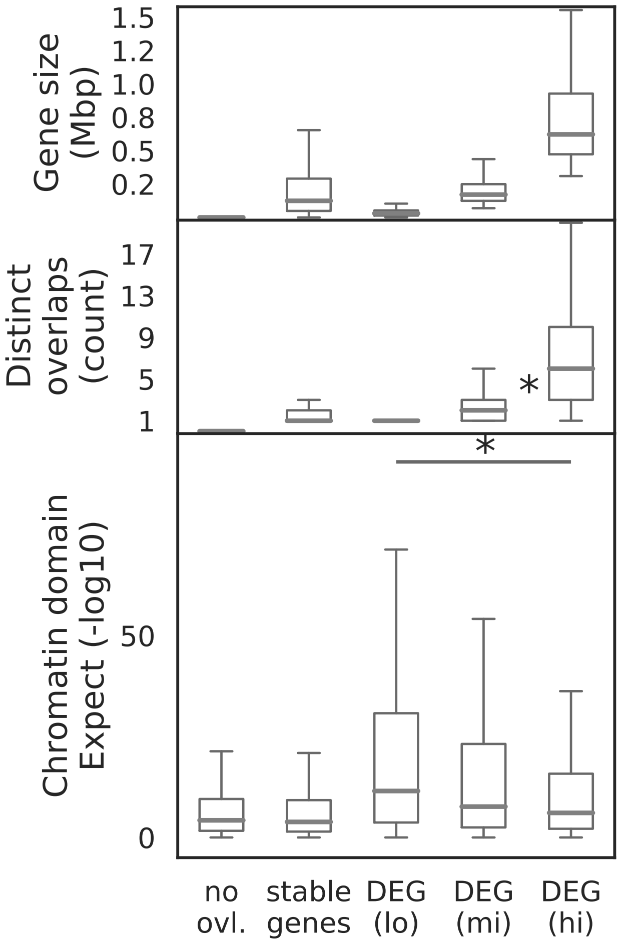

Figure S12: **E-value distribution in DEGs by gene body length**: genes were stratified into four groups based on expression behavior (stable or differential) and their gene body length (shortest 40% [lo], middle 40% [mi] and longest 20% [hi] of DEGs according to gene body length). Bottom: boxplots show distribution of E-values of all DCDs overlapping gene bodies in the respective groups aggregated over all sample comparisons. The no overlap group contains all E-values of DCDs not overlapping any gene. Middle: boxplots show distinct DCD overlaps per gene. Top: boxplots show gene body length distribution of all genes in the respective group. Differences in magnitude of E-values were assessed with a two-sided Mann-Whitney-U test and considered significant (*) with $p < 0.01$.

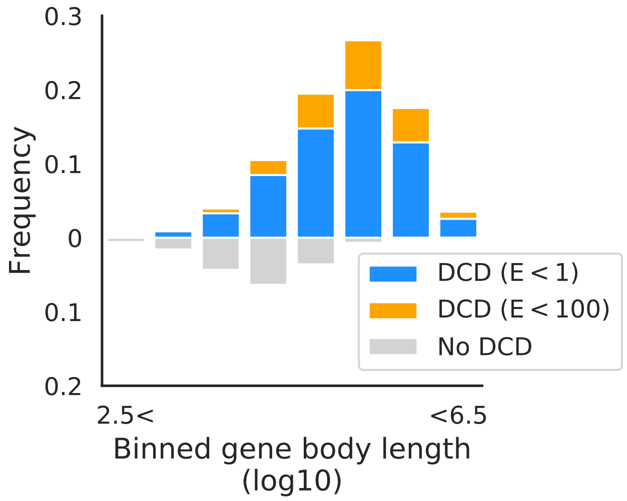

Figure S13: **Relaxing E-value threshold does not improve detection of short DEGs**: all DEGs for all six comparisons were binned based on their gene body size (x-axis) and classified based on overlapping DCDs in their gene body (y-axis). DCDs were called with the default threshold of E $<$ 1 (blue) and with a relaxed threshold of E $<$ 100 (orange).

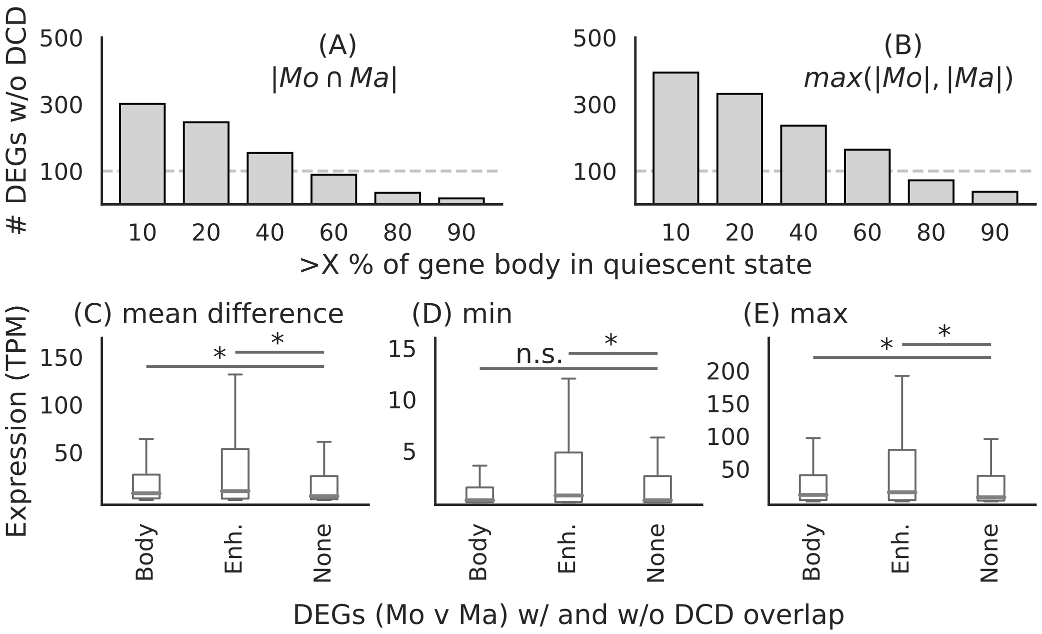

Figure S14: **Uninformative chromatin state in gene bodies and moderate changes in expression complicate DEG recovery**: (top) DEGs were binned according to the fraction of gene body covered with the background "quiescent" chromatin state (x-axis). (A) Height of bars depicts number of genes in intersection between monocyte and macrophage samples. (B) Height of bars depicts maximal number of genes either from monocyte or from macrophage samples. (bottom) DEGs were stratified according to DCD overlap in gene body/promoter (Body), or in at least one enhancer (Enh.) or no DCD overlap (None). Box plots show distribution of gene expression values for absolute mean differences (C) between monocyte and macrophage samples, and for minimal expression (D) and for maximal expression (E) in any sample. Differences in magnitude were assessed using a two-sided Mann-Whitney-U test and considered significant "*" at $p < 0.01$ and not significant (n.s.) otherwise.

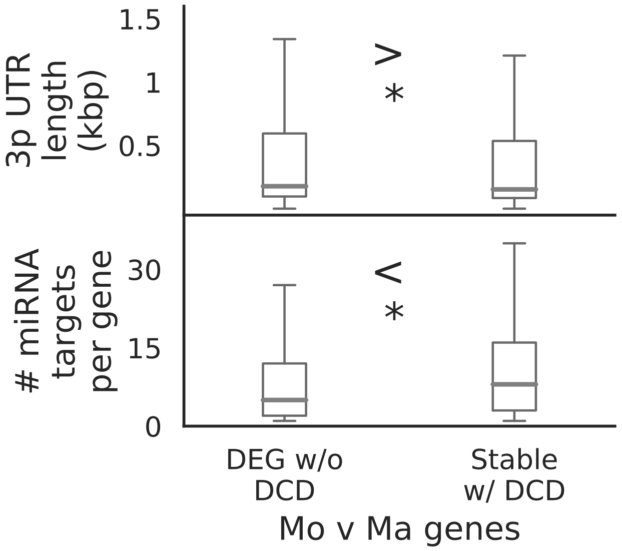

Figure S15: **Difference in annotated miRNA targets and 3p UTR length**: genes overlapping a DCD in their gene body were split into two groups based on expression behavior (stable or differential) for the monocyte to macrophage comparison. (top) box plots show distribution of 3p UTR length as annotated in Ensembl v78 for the genes in the respective groups. (bottom) box plots show distribution of number of annotated miRNA targets per genes in the respective groups (TargetScan v7.2). Differences in magnitude between the two groups were assessed with a one-sided Mann-Whitney-U test (alternative less or greater as indicated) and considered significant "*" at $p < 0.01$.

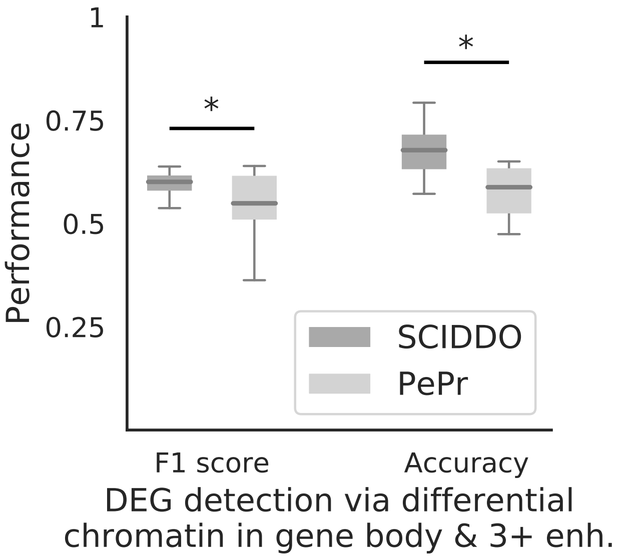

Figure S16: **SCIDDO shows more stable performance at detecting DEGs**: box plots depict SCIDDO's and PePr's (light grey) performance of detecting DEGs quantified as F1 score (left) and as accuracy (right). Performance values are summarized over all sample group comparisons and for different thresholds on gene expression fold change (0.5, 1, 2 and 4) and on adjusted p-values (0.1, 0.05, 0.01 and 0.001) computed with DESeq2 to call DEGs. At least one DCD/differential H3K36me3 peak (PePr) in the gene body and at least three DCDs/differential H3K27ac peaks (PePr) in gene-associated enhancers were required for a DEG to be considered detected on the chromatin level. Differences in performance were assessed with a one-sided Mann-Whitney-U test and considered significant "*" at $p < 0.01$

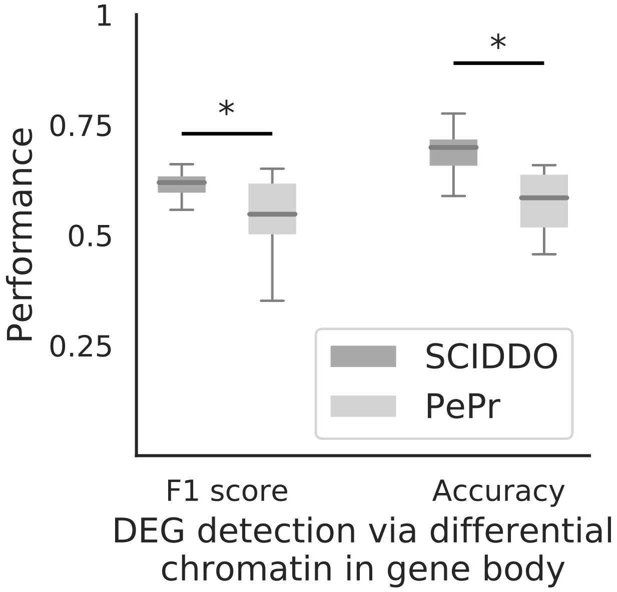

Figure S17: **SCIDDO shows more stable performance at detecting DEGs**: box plots depict SCIDDO's and PePr's (light grey) performance of detecting DEGs quantified as F1 score (left) and as accuracy (right). Performance values are summarized over all sample group comparisons and for different thresholds on gene expression fold change (0.5, 1, 2 and 4) and on adjusted p-values (0.1, 0.05, 0.01 and 0.001) computed with DESeq2 to call DEGs. At least one DCD/differential H3K36me3 peak (PePr) in the gene body was required for a DEG to be considered detected on the chromatin level. The quiescent chromatin state was not treated as "not differential" by default in the SCIDDO analysis. Differences in performance were assessed with a one-sided Mann-Whitney-U test and considered significant "*" at $p < 0.01$

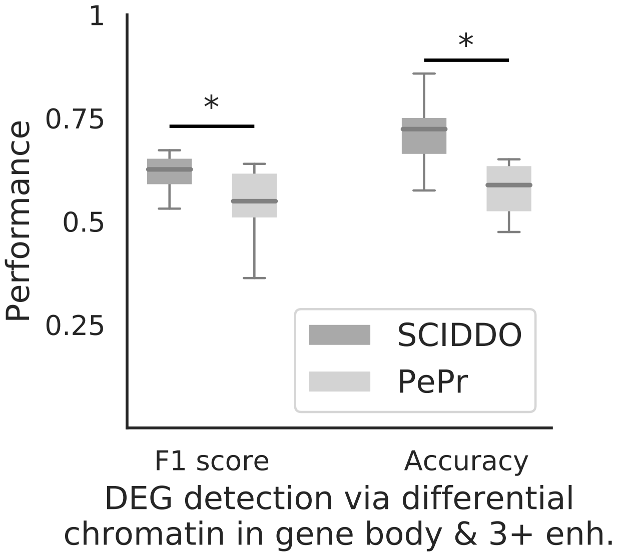

Figure S18: **SCIDDO shows more stable performance at detecting DEGs**: box plots depict SCIDDO's and PePr's (light grey) performance of detecting DEGs quantified as F1 score (left) and as accuracy (right). Performance values are summarized over all sample group comparisons and for different thresholds on gene expression fold change (0.5, 1, 2 and 4) and on adjusted p-values (0.1, 0.05, 0.01 and 0.001) computed with DESeq2 to call DEGs. At least one DCD/differential H3K36me3 peak (PePr) in the gene body and at least three DCDs/differential H3K27ac peaks (PePr) in gene-associated enhancers were required for a DEG to be considered detected on the chromatin level. The quiescent chromatin state was not treated as "not differential" by default in the SCIDDO analysis. Differences in performance were assessed with a one-sided Mann-Whitney-U test and considered significant "*" at $p < 0.01$

4 Supplementary Tables

ID	DEEP sample	donor	histone mark	filename
HG-1	01_HepG2_LiHG_Ct1	A	H3K27ac	01_HepG2_LiHG_Ct1_H3K27ac_S_1.GALv2.20150422.grch38.bam
HG-1	01_HepG2_LiHG_Ct1	A	H3K27me3	01_HepG2_LiHG_Ct1_H3K27me3_S_1.bwamem.20170818.grch38.bam
HG-1	01_HepG2_LiHG_Ct1	A	H3K36me3	01_HepG2_LiHG_Ct1_H3K36me3_S_1.GALv2.20150422.grch38.bam
HG-1	01_HepG2_LiHG_Ct1	A	H3K4me1	01_HepG2_LiHG_Ct1_H3K4me1_S_1.bwamem.20170818.bam
HG-1	01_HepG2_LiHG_Ct1	A	H3K4me3	01_HepG2_LiHG_Ct1_H3K4me3_S_1.bwamem.20170818.bam
HG-1	01_HepG2_LiHG_Ct1	A	H3K9me3	01_HepG2_LiHG_Ct1_H3K9me3_S_1.GALv2.20150422.grch38.bam
HG-1	01_HepG2_LiHG_Ct1	A	Input	01_HepG2_LiHG_Ct1_Input_S_1.GALv2.20150422.grch38.bam
HG-2	01_HepG2_LiHG_Ct2	B	H3K27ac	01_HepG2_LiHG_Ct2_H3K27ac_F_1.bwamem.20170812.bam
HG-2	01_HepG2_LiHG_Ct2	B	H3K27me3	01_HepG2_LiHG_Ct2_H3K27me3_F_1.bwamem.20170812.grch38.bam
HG-2	01_HepG2_LiHG_Ct2	B	H3K36me3	01_HepG2_LiHG_Ct2_H3K36me3_F_1.bwamem.20170812.grch38.bam
HG-2	01_HepG2_LiHG_Ct2	B	H3K4me1	01_HepG2_LiHG_Ct2_H3K4me1_F_1.bwamem.20170812.bam
HG-2	01_HepG2_LiHG_Ct2	B	H3K4me3	01_HepG2_LiHG_Ct2_H3K4me3_F_1.bwamem.20170812.bam
HG-2	01_HepG2_LiHG_Ct2	B	H3K9me3	01_HepG2_LiHG_Ct2_H3K9me3_F_1.bwamem.20170812.bam
HG-2	01_HepG2_LiHG_Ct2	B	Input	01_HepG2_LiHG_Ct2_Input_F_1.bwamem.20170812.bam
He-2	41_Hf02_LiHe_Ct	C	H3K27ac	41_Hf02_LiHe_Ct_H3K27ac_F_1.bwamem.20170812.bam
He-2	41_Hf02_LiHe_Ct	C	H3K27me3	41_Hf02_LiHe_Ct_H3K27me3_F_1.bwamem.20170812.bam
He-2	41_Hf02_LiHe_Ct	C	H3K36me3	41_Hf02_LiHe_Ct_H3K36me3_F_1.bwamem.20170812.bam
He-2	41_Hf02_LiHe_Ct	C	H3K4me1	41_Hf02_LiHe_Ct_H3K4me1_F_1.bwamem.20170812.bam
He-2	41_Hf02_LiHe_Ct	C	H3K4me3	41_Hf02_LiHe_Ct_H3K4me3_F_1.bwamem.20170812.bam
He-2	41_Hf02_LiHe_Ct	C	H3K9me3	41_Hf02_LiHe_Ct_H3K9me3_F_1.bwamem.20170812.bam
He-2	41_Hf02_LiHe_Ct	C	Input	41_Hf02_LiHe_Ct_Input_F_1.bwamem.20170812.bam
He-3	41_Hf03_LiHe_Ct	D	H3K27ac	41_Hf03_LiHe_Ct_H3K27ac_F_1.bwamem.20170811.bam
He-3	41_Hf03_LiHe_Ct	D	H3K27me3	41_Hf03_LiHe_Ct_H3K27me3_F_1.bwamem.20170811.bam
He-3	41_Hf03_LiHe_Ct	D	H3K36me3	41_Hf03_LiHe_Ct_H3K36me3_F_1.bwamem.20170811.bam
He-3	41_Hf03_LiHe_Ct	D	H3K4me1	41_Hf03_LiHe_Ct_H3K4me1_F_1.bwamem.20170811.bam
He-3	41_Hf03_LiHe_Ct	D	H3K4me3	41_Hf03_LiHe_Ct_H3K4me3_F_1.bwamem.20170811.bam
He-3	41_Hf03_LiHe_Ct	D	H3K9me3	41_Hf03_LiHe_Ct_H3K9me3_F_1.bwamem.20170812.bam
He-3	41_Hf03_LiHe_Ct	D	Input	41_Hf03_LiHe_Ct_Input_F_1.bwamem.20170812.bam
Ma-3	43_Hm03_BlMa_Ct	E	H3K27ac	43_Hm03_BlMa_Ct_H3K27ac_F_1.bwamem.20170811.bam
Ma-3	43_Hm03_BlMa_Ct	E	H3K27me3	43_Hm03_BlMa_Ct_H3K27me3_F_1.bwamem.20170812.bam
Ma-3	43_Hm03_BlMa_Ct	E	H3K36me3	43_Hm03_BlMa_Ct_H3K36me3_F_1.bwamem.20170811.bam
Ma-3	43_Hm03_BlMa_Ct	E	H3K4me1	43_Hm03_BlMa_Ct_H3K4me1_F_1.bwamem.20170811.bam
Ma-3	43_Hm03_BlMa_Ct	E	H3K4me3	43_Hm03_BlMa_Ct_H3K4me3_F_1.bwamem.20170811.bam
Ma-3	43_Hm03_BlMa_Ct	E	H3K9me3	43_Hm03_BlMa_Ct_H3K9me3_F_1.bwamem.20170811.bam
Ma-3	43_Hm03_BlMa_Ct	E	Input	43_Hm03_BlMa_Ct_Input_F_1.bwamem.20170811.bam
Ma-5	43_Hm05_BlMa_Ct	F	H3K27ac	43_Hm05_BlMa_Ct_H3K27ac_F_1.bwamem.20170812.bam
Ma-5	43_Hm05_BlMa_Ct	F	H3K27me3	43_Hm05_BlMa_Ct_H3K27me3_F_1.bwamem.20170812.bam
Ma-5	43_Hm05_BlMa_Ct	F	H3K36me3	43_Hm05_BlMa_Ct_H3K36me3_F_1.bwamem.20170811.bam
Ma-5	43_Hm05_BlMa_Ct	F	H3K4me1	43_Hm05_BlMa_Ct_H3K4me1_F_1.bwamem.20170811.bam
Ma-5	43_Hm05_BlMa_Ct	F	H3K4me3	43_Hm05_BlMa_Ct_H3K4me3_F_1.bwamem.20170811.bam
Ma-5	43_Hm05_BlMa_Ct	F	H3K9me3	43_Hm05_BlMa_Ct_H3K9me3_F_1.bwamem.20170812.bam
Ma-5	43_Hm05_BlMa_Ct	F	Input	43_Hm05_BlMa_Ct_Input_F_1.bwamem.20170812.bam
Mo-1	43_Hm01_BlMo_Ct	G	H3K27ac	43_Hm01_BlMo_Ct_H3K27ac_F_1.bwamem.20170812.bam
Mo-1	43_Hm01_BlMo_Ct	G	H3K27me3	43_Hm01_BlMo_Ct_H3K27me3_F_1.bwamem.20170812.bam
Mo-1	43_Hm01_BlMo_Ct	G	H3K36me3	43_Hm01_BlMo_Ct_H3K36me3_F_1.bwamem.20170812.bam
Mo-1	43_Hm01_BlMo_Ct	G	H3K4me1	43_Hm01_BlMo_Ct_H3K4me1_F_1.bwamem.20170812.bam
Mo-1	43_Hm01_BlMo_Ct	G	H3K4me3	43_Hm01_BlMo_Ct_H3K4me3_F_1.bwamem.20170812.bam
Mo-1	43_Hm01_BlMo_Ct	G	H3K9me3	43_Hm01_BlMo_Ct_H3K9me3_F_1.bwamem.20170812.bam
Mo-1	43_Hm01_BlMo_Ct	G	Input	43_Hm01_BlMo_Ct_Input_F_1.bwamem.20170812.bam
Mo-3	43_Hm03_BlMo_Ct	E	H3K27ac	43_Hm03_BlMo_Ct_H3K27ac_F_1.bwamem.20170811.bam
Mo-3	43_Hm03_BlMo_Ct	E	H3K27me3	43_Hm03_BlMo_Ct_H3K27me3_F_1.bwamem.20170811.bam
Mo-3	43_Hm03_BlMo_Ct	E	H3K36me3	43_Hm03_BlMo_Ct_H3K36me3_F_1.bwamem.20170811.bam
Mo-3	43_Hm03_BlMo_Ct	E	H3K4me1	43_Hm03_BlMo_Ct_H3K4me1_F_1.bwamem.20170811.bam
Mo-3	43_Hm03_BlMo_Ct	E	H3K4me3	43_Hm03_BlMo_Ct_H3K4me3_F_1.bwamem.20170811.bam
Mo-3	43_Hm03_BlMo_Ct	E	H3K9me3	43_Hm03_BlMo_Ct_H3K9me3_F_1.bwamem.20170812.bam
Mo-3	43_Hm03_BlMo_Ct	E	Input	43_Hm03_BlMo_Ct_Input_F_1.bwamem.20170812.bam
Mo-5	43_Hm05_BlMo_Ct	F	H3K27ac	43_Hm05_BlMo_Ct_H3K27ac_F_1.bwamem.20170811.bam
Mo-5	43_Hm05_BlMo_Ct	F	H3K27me3	43_Hm05_BlMo_Ct_H3K27me3_F_1.bwamem.20170812.bam
Mo-5	43_Hm05_BlMo_Ct	F	H3K36me3	43_Hm05_BlMo_Ct_H3K36me3_F_1.bwamem.20170812.bam
Mo-5	43_Hm05_BlMo_Ct	F	H3K4me1	43_Hm05_BlMo_Ct_H3K4me1_F_1.bwamem.20170811.bam
Mo-5	43_Hm05_BlMo_Ct	F	H3K4me3	43_Hm05_BlMo_Ct_H3K4me3_F_1.bwamem.20170811.bam
Mo-5	43_Hm05_BlMo_Ct	F	H3K9me3	43_Hm05_BlMo_Ct_H3K9me3_F_1.bwamem.20170812.bam
Mo-5	43_Hm05_BlMo_Ct	F	Input	43_Hm05_BlMo_Ct_Input_F_1.bwamem.20170812.bam

Table S1: Overview of DEEP histone data used to generate chromatin state segmentation maps. Unique donor labels A to F have been assigned from top to bottom in lexicographical order of the ID. Access to the data files listed here is restricted due to patient privacy. Access to the raw data can be requested under www.ebi.ac.uk/ega/dacs/EGAC00001000179. Processed datasets are publicly available as IHEC trackhubs under epigenomesportal.ca/ihec.

ID	DEEP sample	donor	filename
HG-1	01_HepG2_LiHG_Ct1	A	01_HepG2_LiHG_Ct1_mRNA_K_1_ATCACG_L001_R1
HG-1	01_HepG2_LiHG_Ct1	A	01_HepG2_LiHG_Ct1_mRNA_K_1_ATCACG_L001_R2
HG-2	01_HepG2_LiHG_Ct2	B	01_HepG2_LiHG_Ct2_mRNA_K_1_GAGTGG_L002_R1
HG-2	01_HepG2_LiHG_Ct2	B	01_HepG2_LiHG_Ct2_mRNA_K_1_GAGTGG_L002_R2
He-2	41_Hf02_LiHe_Ct	C	41_Hf02_LiHe_Ct_mRNA_K_1_GAGTGG_L005_R1
He-2	41_Hf02_LiHe_Ct	C	41_Hf02_LiHe_Ct_mRNA_K_1_GAGTGG_L005_R2
He-2	41_Hf02_LiHe_Ct	C	41_Hf02_LiHe_Ct_mRNA_K_1_GAGTGG_L006_R1
He-2	41_Hf02_LiHe_Ct	C	41_Hf02_LiHe_Ct_mRNA_K_1_GAGTGG_L006_R2
He-2	41_Hf02_LiHe_Ct	C	41_Hf02_LiHe_Ct_mRNA_K_1_GAGTGG_L007_R1
He-2	41_Hf02_LiHe_Ct	C	41_Hf02_LiHe_Ct_mRNA_K_1_GAGTGG_L007_R2
He-2	41_Hf02_LiHe_Ct	C	41_Hf02_LiHe_Ct_mRNA_K_1_GAGTGG_L008_R1
He-2	41_Hf02_LiHe_Ct	C	41_Hf02_LiHe_Ct_mRNA_K_1_GAGTGG_L008_R2
He-3	41_Hf03_LiHe_Ct	D	41_Hf03_LiHe_Ct_mRNA_K_1_CGATGT_L003_R1
He-3	41_Hf03_LiHe_Ct	D	41_Hf03_LiHe_Ct_mRNA_K_1_CGATGT_L003_R2
Ma-3	43_Hm03_BlMa_Ct	E	43_Hm03_BlMa_Ct_mRNA_M_1_ACTTGA_L005_R1
Ma-3	43_Hm03_BlMa_Ct	E	43_Hm03_BlMa_Ct_mRNA_M_1_ACTTGA_L005_R2
Ma-3	43_Hm03_BlMa_Ct	E	43_Hm03_BlMa_Ct_mRNA_M_1_ACTTGA_L006_R1
Ma-3	43_Hm03_BlMa_Ct	E	43_Hm03_BlMa_Ct_mRNA_M_1_ACTTGA_L006_R2
Ma-3	43_Hm03_BlMa_Ct	E	43_Hm03_BlMa_Ct_mRNA_M_1_ACTTGA_L007_R1
Ma-3	43_Hm03_BlMa_Ct	E	43_Hm03_BlMa_Ct_mRNA_M_1_ACTTGA_L007_R2
Ma-3	43_Hm03_BlMa_Ct	E	43_Hm03_BlMa_Ct_mRNA_M_1_ACTTGA_L008_R1
Ma-3	43_Hm03_BlMa_Ct	E	43_Hm03_BlMa_Ct_mRNA_M_1_ACTTGA_L008_R2
Ma-5	43_Hm05_BlMa_Ct	F	43_Hm05_BlMa_Ct_mRNA_M_1_TTAGGC_L001_R1
Ma-5	43_Hm05_BlMa_Ct	F	43_Hm05_BlMa_Ct_mRNA_M_1_TTAGGC_L001_R2
Ma-5	43_Hm05_BlMa_Ct	F	43_Hm05_BlMa_Ct_mRNA_M_1_TTAGGC_L002_R1
Ma-5	43_Hm05_BlMa_Ct	F	43_Hm05_BlMa_Ct_mRNA_M_1_TTAGGC_L002_R2
Ma-5	43_Hm05_BlMa_Ct	F	43_Hm05_BlMa_Ct_mRNA_M_1_TTAGGC_L003_R1
Ma-5	43_Hm05_BlMa_Ct	F	43_Hm05_BlMa_Ct_mRNA_M_1_TTAGGC_L003_R2
Ma-5	43_Hm05_BlMa_Ct	F	43_Hm05_BlMa_Ct_mRNA_M_1_TTAGGC_L004_R1
Ma-5	43_Hm05_BlMa_Ct	F	43_Hm05_BlMa_Ct_mRNA_M_1_TTAGGC_L004_R2
Mo-1	43_Hm01_BlMo_Ct	G	43_Hm01_BlMo_Ct_mRNA_M_1_AGTCAA_L005_R1
Mo-1	43_Hm01_BlMo_Ct	G	43_Hm01_BlMo_Ct_mRNA_M_1_AGTCAA_L005_R2
Mo-1	43_Hm01_BlMo_Ct	G	43_Hm01_BlMo_Ct_mRNA_M_1_AGTCAA_L006_R1
Mo-1	43_Hm01_BlMo_Ct	G	43_Hm01_BlMo_Ct_mRNA_M_1_AGTCAA_L006_R2
Mo-1	43_Hm01_BlMo_Ct	G	43_Hm01_BlMo_Ct_mRNA_M_1_AGTCAA_L007_R1
Mo-1	43_Hm01_BlMo_Ct	G	43_Hm01_BlMo_Ct_mRNA_M_1_AGTCAA_L007_R2
Mo-1	43_Hm01_BlMo_Ct	G	43_Hm01_BlMo_Ct_mRNA_M_1_AGTCAA_L008_R1
Mo-1	43_Hm01_BlMo_Ct	G	43_Hm01_BlMo_Ct_mRNA_M_1_AGTCAA_L008_R2
Mo-3	43_Hm03_BlMo_Ct	E	43_Hm03_BlMo_Ct_mRNA_M_1_ATCACG_L005_R1
Mo-3	43_Hm03_BlMo_Ct	E	43_Hm03_BlMo_Ct_mRNA_M_1_ATCACG_L005_R2
Mo-3	43_Hm03_BlMo_Ct	E	43_Hm03_BlMo_Ct_mRNA_M_1_ATCACG_L006_R1
Mo-3	43_Hm03_BlMo_Ct	E	43_Hm03_BlMo_Ct_mRNA_M_1_ATCACG_L006_R2
Mo-3	43_Hm03_BlMo_Ct	E	43_Hm03_BlMo_Ct_mRNA_M_1_ATCACG_L007_R1
Mo-3	43_Hm03_BlMo_Ct	E	43_Hm03_BlMo_Ct_mRNA_M_1_ATCACG_L007_R2
Mo-3	43_Hm03_BlMo_Ct	E	43_Hm03_BlMo_Ct_mRNA_M_1_ATCACG_L008_R1
Mo-3	43_Hm03_BlMo_Ct	E	43_Hm03_BlMo_Ct_mRNA_M_1_ATCACG_L008_R2
Mo-5	43_Hm05_BlMo_Ct	F	43_Hm05_BlMo_Ct_mRNA_M_1_ATCACG_L001_R1
Mo-5	43_Hm05_BlMo_Ct	F	43_Hm05_BlMo_Ct_mRNA_M_1_ATCACG_L001_R2
Mo-5	43_Hm05_BlMo_Ct	F	43_Hm05_BlMo_Ct_mRNA_M_1_ATCACG_L002_R1
Mo-5	43_Hm05_BlMo_Ct	F	43_Hm05_BlMo_Ct_mRNA_M_1_ATCACG_L002_R2
Mo-5	43_Hm05_BlMo_Ct	F	43_Hm05_BlMo_Ct_mRNA_M_1_ATCACG_L003_R1
Mo-5	43_Hm05_BlMo_Ct	F	43_Hm05_BlMo_Ct_mRNA_M_1_ATCACG_L003_R2
Mo-5	43_Hm05_BlMo_Ct	F	43_Hm05_BlMo_Ct_mRNA_M_1_ATCACG_L004_R1
Mo-5	43_Hm05_BlMo_Ct	F	43_Hm05_BlMo_Ct_mRNA_M_1_ATCACG_L004_R2

Table S2: Overview of DEEP expression data used used to compute differentially expressed genes. Unique donor labels A to F have been assigned from top to bottom in lexicographical order of the ID. Access to the data files listed here is restricted due to patient privacy. Access to the raw data can be requested under www.ebi.ac.uk/ega/dacs/EGAC00001000179. Processed datasets are publicly available as IHEC trackhubs under epigenomesportal.ca/ihec.

number	mnemonic	description
1	TssA	Active TSS
2	TssFlnk	Flanking TSS
3	TssFlnkU	Flanking TSS upstream
4	TssFlnkD	Flanking TSS downstream
5	Tx	Strong transcription
6	TxWk	Weak transcription
7	EnhG1	Genic enhancer1
8	EnhG2	Genic enhancer2
9	EnhA1	Active enhancer 1
10	EnhA2	Active enhancer 2
11	EnhWk	Weak enhancer
12	ZNF/Rpts	ZNF genes & repeats
13	Het	Heterochromatin
14	TssBiv	Bivalent/Poised TSS
15	EnhBiv	Bivalent enhancer
16	ReprPC	Repressed PolyComb
17	WkReprPC	Weak repressed PolyComb
18	Quies	Quiescent/Low

Table S3: State numbers, mnemonics and concise descriptions of the chromatin states of the ChromHMM CMM18 model as provided by the REMC under egg2.wustl.edu/roadmap/web_portal/chr_state_learning.html

command	cores	samples	runtime (min)
convert	7	9	< 3
stats	7	9	< 1
score	1	n/a	< 1
scan	15	2 v 2	< 4
scan	15	4 v 5	< 7

Table S4: Runtime (in minutes of wall clock time) of individual SCIDDO commands executed in order from top to bottom to perform the differential analysis. The runtime for the `scan` command refers to a single comparison of two versus two samples. The last `scan` command is provided as an example of the scaling behavior of SCIDDO (scanning for differential chromatin domains between the four liver and the five blood samples in the dataset). Note that the runtime includes I/O.

group1	group2	Spearman's ρ	unique regions %
HG	He	0.67 (0.06)	10.85 (3.14)
HG	Ma	0.7 (0.04)	7.51 (3.6)
HG	Mo	0.73 (0.04)	3.92 (1.68)
He	Ma	0.68 (0.04)	5.99 (1.35)
He	Mo	0.7 (0.03)	3.27 (0.39)
Mo	Ma	0.7 (0.04)	17.68 (3.17)

Table S5: Average Spearman correlation of E–values of all overlapping candidate regions identified in individual replicate comparisons. Rightmost column indicates the average percentage of unique candidate regions per comparison. Values in parentheses give +/- 1 standard deviation for the respective statistic.

5 Supplementary References

References

Vikram Agarwal, George W Bell, Jin-Wu Nam, and David P Bartel. Predicting effective microrna target sites in mammalian mrnas. *eLife*, 4:e05005, 2015. ISSN 2050-084X. doi: 10.7554/eLife.05005.

Timothy Bailey, Pawel Krajewski, Istvan Ladunga, Celine Lefebvre, Qunhua Li, Tao Liu, et al. Practical guidelines for the comprehensive analysis of ChIP-seq data. *PLoS computational biology*, 9(11), 2013. doi: 10.1371/journal.pcbi.1003326.

Antoine Coulon, Carson C. Chow, Robert H. Singer, and Daniel R. Larson. Eukaryotic transcriptional dynamics: from single molecules to cell populations. *Nature Reviews Genetics*, 14(8):572–584, 2013. ISSN 1471-0056. doi: 10.1038/nrg3484.

Simon Fishilevich, Ron Nudel, Noa Rappaport, Rotem Hadar, Inbar Plaschkes, Tsippi Iny Stein, Naomi Rosen, Asher Kohn, Michal Twik, Marilyn Safran, Doron Lancet, and Dana Cohen. GeneHancer: genome-wide integration of enhancers and target genes in GeneCards. *Database*, 2017(1):1665–1680, 2017. ISSN 1758-0463. doi: 10.1093/database/bax028.

Jennifer Harrow, Adam Frankish, Jose M Gonzalez, Electra Tapanari, Mark Diekhans, Felix Kokocinski, et al. GENCODE: the reference human genome annotation for The ENCODE Project. *Genome Research*, 22(9):1760–74, 2012. doi: 10.1101/gr.135350.111.

Steven R Head, H Kiyomi Komori, Sarah A LaMere, Thomas Whisenant, Filip Van Nieuwerburgh, Daniel R Salomon, and Phillip Ordoukhanian. Library construction for next-generation sequencing: overviews and challenges. *BioTechniques*, 56(2), 2014. doi: 10.2144/000114133.

S Karlin and S F Altschul. Applications and statistics for multiple high-scoring segments in molecular sequences. *Proceedings of the National Academy of Sciences of the United States of America*, 90(12):5873–5877, 1993. ISSN 0027-8424. doi: 10.1073/pnas.90.12.5873.

Samuel Karlin, Amir Dembo, and Tsutomu Kawabata. Statistical Composition of High-Scoring Segments from Molecular Sequences. *The Annals of Statistics*, 18(2):571–581, 1990. ISSN 0090-5364. doi: 10.1214/aos/1176347616.

Tae Hoon Kim and Bing Ren. An all-round view of eukaryotic transcription. *Genome biology*, 7(7):323, 2006. ISSN 1465-6914. doi: 10.1186/gb-2006-7-7-323.

Stephen G Landt, Georgi K Marinov, Anshul Kundaje, Pouya Kheradpour, Florencia Pauli, Serafim Batzoglou, et al. ChIP-seq guidelines and practices of the ENCODE

and modENCODE consortia. *Genome research*, 22(9):1813–31, 2012. doi: 10.1101/gr.136184.111.

Michael I Love, Wolfgang Huber, and Simon Anders. Moderated estimation of fold change and dispersion for RNA-seq data with DESeq2. *Genome Biology*, 15 (12):550, 2014. ISSN 1474-760X. doi: 10.1186/s13059-014-0550-8. URL http://biorxiv.org/content/biorxiv/early/2014/02/19/002832.full.pdfhttp://genomebiology.com/2014/15/12/550http://genomebiology.biomedcentral.com/articles/10.1186/s13059-014-0550-8.

George Orphanides and Danny Reinberg. A unified theory of gene expression. *Cell*, 108 (4):439–451, 2002.

Rob Patro, Geet Duggal, Michael I. Love, Rafael A. Irizarry, and Carl Kingsford. Salmon provides fast and bias-aware quantification of transcript expression. *Nature Methods*, 14(4):417–419, 2017. ISSN 1548-7091. doi: 10.1038/nmeth.4197. URL http://dx.doi.org/10.1038/nmeth.4197http://www.nature.com/articles/nmeth.4197.

Madapura M Pradeepa. Causal role of histone acetylations in enhancer function. *Transcription*, 8(1):40–47, 2017. ISSN 2154-1264. doi: 10.1080/21541264.2016.1253529.

Aaron R. Quinlan and Ira M. Hall. BEDTools: a flexible suite of utilities for comparing genomic features. *Bioinformatics*, 26(6):841–842, 2010. doi: 10.1093/bioinformatics/btq033.

Charlotte Soneson, Michael I. Love, and Mark D. Robinson. Differential analyses for RNA-seq: transcript-level estimates improve gene-level inferences. *F1000Research*, 4 (2):1521, 2016. doi: 10.12688/f1000research.7563.2. URL https://f1000research.com/articles/4-1521/v2.

Artem Tarasov, Albert J. Vilella, Edwin Cuppen, Isaac J. Nijman, and Pjotr Prins. Sambamba: fast processing of NGS alignment formats. *Bioinformatics*, 31(12):2032–2034, 2015. ISSN 1367-4803. doi: 10.1093/bioinformatics/btv098.

The ENCODE Project Consortium, Ian Dunham, Anshul Kundaje, Shelley F Aldred, Patrick J Collins, Carrie A Davis, et al. An integrated encyclopedia of DNA elements in the human genome. *Nature*, 489(7414):57–74, 2012. doi: 10.1038/nature11247. URL http://www.pubmedcentral.nih.gov/articlerender.fcgi?artid=3439153{&}tool=pmcentrez{&}rendertype=abstract.

The Roadmap Epigenomics Consortium, Anshul Kundaje, Wouter Meuleman, Jason Ernst, Misha Bilenky, Angela Yen, et al. Integrative analysis of 111 reference human epigenomes. *Nature*, 518(7539):317–330, 2015. doi: 10.1038/nature14248. URL http://www.nature.com/doifinder/10.1038/nature14248.

Stefan Wallner, Christopher Schröder, Elsa Leitão, Tea Berulava, Claudia Haak, Daniela Beißer, et al. Epigenetic dynamics of monocyte-to-macrophage differentiation. *Epigenetics and Chromatin*, 9(1):1–17, 2016. doi: 10.1186/s13072-016-0079-z.

Daniel R Zerbino, Steven P Wilder, Nathan Johnson, Thomas Juettemann, and Paul R Flicek. The Ensembl Regulatory Build. *Genome Biology*, 16(1):56, 2015. doi: 10.1186/s13059-015-0621-5. URL http://genomebiology.com/2015/16/1/56.

Yanxiao Zhang, Y.-H. Lin, Timothy D Johnson, Laura S Rozek, and Maureen A Sartor. PePr: a peak-calling prioritization pipeline to identify consistent or differential peaks from replicated ChIP-Seq data. *Bioinformatics*, 30(18):2568–2575, 2014. doi: 10.1093/bioinformatics/btu372.
